# BIM-Supported green building adoption in the construction industry

**DOI:** 10.1371/journal.pone.0356894

**Published:** 2026-09-15

**Authors:** Khaled A. Alrasheed, Abdulaziz Alotaibi, Abdullah O. Baarimah, Aawag Moshen Alawag, Hamad Almujibah, Madhusudhan Bangalore Ramu

**Affiliations:** 1 College of Engineering & Petroleum, Kuwait University, Kuwait; 2 Department of Civil Engineering, Faculty of Engineering, Islamic University of Madinah, Madinah, Saudi Arabia; 3 Department of Civil and Construction Engineering, College of Engineering, A’Sharqiyah University, Ibra, Oman; 4 Faculty of Engineering and Information Technology, Taiz University, Taiz, Yemen; 5 Department of Civil Engineering, College of Engineering, Taif University, Taif City, Saudi Arabia; 6 Department of Civil and Construction Engineering, College of Engineering, A’Sharqiyah University, Ibra, Oman; Longgang Otorhinolaryngology Hospital & Shenzhen Key Laboratory of Otorhinolaryngology, Shenzhen Institute of Otorhinolaryngology, CHINA

## Abstract

The construction industry is under growing pressure to embrace digital technologies that advance sustainable infrastructure and support the transition toward smart cities. Building Information Modeling (BIM) has emerged as a key enabler of green building practices, yet its adoption across construction organizations remains uneven and the factors shaping this adoption are not fully understood. This study investigates the determinants influencing BIM-supported green building adoption and examines its contribution to smart and sustainable city development. Drawing on the Technology–Organization–Environment (TOE) framework and Diffusion of Innovation (DOI) theory, an integrated model was developed to assess the effects of technological competence, top management support, organizational readiness, external support, environmental sustainability orientation, and relative advantage on BIM-supported green building adoption. Survey data were collected from 389 construction professionals, including architects, engineers, project managers, and BIM coordinators, and analyzed using Partial Least Squares Structural Equation Modeling (PLS-SEM) in SmartPLS. The results reveal that technological competence (β = 0.399), top management support (β = 0.378), and relative advantage (β = 0.317) are the strongest drivers of BIM-supported green building adoption, while environmental sustainability orientation, external support, and organizational readiness exert smaller but significant positive effects. More broadly, these determinants of BIM-supported green building adoption are relevant to the wider goal of smart and sustainable city development, given the role of green buildings in enabling data-driven design, lifecycle performance analysis, and environmentally responsible urban decision-making. This study extends technology adoption research by integrating TOE and DOI perspectives within a sustainability-driven construction context offers practical guidance for construction firms and policymakers operating in similar rapidly developing, sustainability-focused markets.

## Introduction

Urban environments are facing unprecedented pressure to accommodate rapid population growth while simultaneously addressing environmental sustainability challenges [1]. The built environment plays a central role in this transformation because buildings account for a substantial proportion of global energy consumption, carbon emissions, and natural resource utilization [2]. As cities expand and infrastructure demands increase, construction activities must evolve toward more environmentally responsible practices that reduce ecological impact while ensuring long term economic and social sustainability [3,4]. In response to these challenges, the concept of green building development has emerged as a critical strategy for promoting sustainable urban growth through energy efficient design, resource conservation, and improved environmental performance across the lifecycle of buildings [5].

Green building is a way of creating construction that limits the negative impact on the environment from construction, through the integration of sustainable design, as well as the efficient use of materials, and other forms of advanced building technology [6,7]. Green buildings are also designed to be more energy-efficient, produce less waste, conserve water, and improve the indoor environment for occupants [8]. Although there are many reasons to pursue green building, including both environmental and financial benefits, the widespread adoption of green building throughout the construction industry has been inconsistent [9,10]. The challenges many businesses have in successfully implementing sustainable business models include the technological barriers they encounter, the lack of effective collaboration among the various stakeholders involved in construction projects, the inability of some organizations to develop or implement new processes and systems (i.e., lack of capability), and the uncertainty about whether the money invested in sustainable activities will provide an acceptable return on investment [11,12]. Therefore, it has become very important for businesses in the construction industry to begin developing digital technologies that can support improved information exchange and collaboration; and that will enable these businesses to better pursue sustainable construction practices [13].

BIM is becoming an important part of the digital transformation of the construction industry by enabling the integration of all the different aspects of a building’s design, construction and operation into one unified digital platform [14,15]. The BIM platform allows for the creation of intelligent building models that contain all the necessary information to create a building; including architectural, structural, mechanical and operational data [16]. Using the BIM platform, stakeholders involved in a building project are able to visually view and understand how the various building components interact, identify potential design conflicts, simulate how a building will perform, and better coordinate their work efforts in comparison to the use of traditional construction methods [17]. By enhancing communication and coordination among architects, engineers, contractors, and project managers through the use of the BIM platform, it helps to improve the efficiency of a construction project and reduce errors associated with construction throughout its lifecycle [18].

BIM’s contribution is far from merely facilitating better coordination and visualization in construction projects; it has an equally important role to play as a basis for sustainability-based decision making throughout the early stages of a project [19]. For example, by using BIM as the basis for analytical tools, the project team can assess building energy usage, daylight penetration, and thermal efficiency as well as the environmental impact prior to commencing construction [20]. Using these simulation capabilities, stakeholders are able to compare different design options and determine which design will have the least negative impacts on the environment, yet maximize building performance [21]. In this way, BIM offers a powerful technological platform for the advancement of green building development and improved sustainability of urban infrastructure systems [22].

The use of BIM to achieve green building practices is a major advantage for sustainable development. However, numerous technological, organizational and environmental factors affect how well an organization will be able to adopt BIM supported green building practices [23,24]. For example, organizations that do not have adequate technological capability, or do not have access to trained personnel and appropriate management systems will not be able to adequately utilize BIM technologies [25]. Organizations will also need to consider their external environment (i.e., local regulatory requirements, industry standards, professional networks) when making decisions about adopting new technology [26]. Organizational willingness to invest in BIM solutions is also influenced by how much individual employees believe they can benefit from using BIM [27]. If employees view BIM as providing them with a distinct advantage over other construction methods (e.g., improved project performance, increased collaboration among team members, environmental sustainability), then those employees will be more willing to incorporate BIM into the work processes of their organization [28].

To provide a structured understanding of technology adoption within organizational environments, theoretical perspectives such as the Technology Organization Environment framework and Diffusion of Innovation theory have been widely applied in construction management research [29]. The Technology Organization Environment (TOE) model provides a way to explain which factors are important for firms to be able to adopt new technologies [30], in addition to explaining why there will be differences in the rate of technology diffusion across firms based on their technical capability, the environment they operate in and internal organization structure/conditions. In contrast, the Diffusion of Innovation Theory provides insight into what makes an individual or group likely to accept and use an innovation, as well as when this is most likely to occur. As such, the TOE model and Diffusion of Innovation Theory provide complementary frameworks with which to understand the complexities involved in the decision-making processes related to the adoption of Building Information Modeling (BIM) within construction organizations [31,32].

Despite the growing body of research on BIM adoption, the literature remains fragmented and, in places, contradictory. Existing studies have examined BIM adoption largely as a general digital-transformation phenomenon, yet far fewer have isolated the determinants of BIM adoption specifically for green building and sustainability purposes, where the drivers may differ from conventional BIM use [33–35]. Moreover, prior TOE- and DOI-based studies report inconsistent findings regarding the relative importance of organizational versus environmental factors: some emphasize internal readiness and leadership as decisive, while others foreground external regulatory and institutional pressures. These inconsistencies are especially pronounced in rapidly developing, sustainability-focused markets, where regulatory frameworks and digital maturity are evolving quickly and where empirical evidence remains scarce. This study addresses these gaps by integrating TOE and DOI perspectives to examine, within a single coherent model, which technological, organizational, and environmental determinants most strongly shape BIM-supported green building adoption in the Saudi construction sector.

Building upon these theoretical foundations, this study aim to investigates the determinants that influence the adoption of BIM supported green building practices within construction organizations indicating the following objectives.

- To identify the technological, organizational, and environmental factors that influence the adoption of BIM supported green building practices within construction organizations.
- To examine how managerial support and organizational readiness facilitate the implementation of BIM based sustainable construction strategies.
- To discuss how BIM-supported green building adoption relates to the broader agenda of smart and sustainable city development.

Smart and sustainable city development is a new direction in urban planning. The new direction will provide ways to use digital technologies; environmentally friendly infrastructure; and data-driven decision-making to improve cities’ ability to recover from disasters (resilience) and be more habitable [36,37]. Sustainable buildings are one part of an urban ecosystem as they help reduce energy usage and greenhouse gases produced; and improve how resources are used [38]. Therefore, BIM-supported green building development has the potential to support smart city development through digital platforms for planning, monitoring and optimizing building performance throughout the urban life cycle [39].

This research contributes to developing body of literature on the digital transformation in sustainable construction by providing insight into the factors that influence the adoption of BIM-enabled green building projects from an integrated theoretical perspective. It is anticipated that these findings will be useful to organizations involved in the construction industry who seek to improve their digital competency, and may also offer useful reference points for policymakers and urban planners considering how firm-level digital adoption relates to broader sustainability goals, although this study’s evidence base is limited to construction professionals. By providing a greater awareness of the drivers behind the adoption of BIM-enabled sustainable practices, this research demonstrates how digital technology can assist with the environmental responsibility and technological advancements associated with transitioning toward urban development.

## Literature review and theoretical foundation

### Theoretical foundation of the study

To understand the acceptance of digital technology in the construction industry it is important to take a comprehensive view of the factors influencing its use. Dual theoretical frameworks are used for this purpose. In order to determine which factors influence the acceptance of BIM as an innovation supporting the delivery of green buildings, the authors will be using a combination of two theoretical models [40]. The first framework focus on three aspects of organization and technological innovation. It consider technological capability; organizational readiness; and environmental influences (Technology Organization Environment). The second framework look at the decision-making process regarding the adoption of new innovative technologies by considering the degree of innovativeness of those technologies (Diffusion of Innovation Theory).

The Technology Organization Environment (TOE) framework uses three contextual factors that help explain how organizations make decisions about adopting innovation. The technological environment represents the availability of innovative technologies and their ability to support implementing them [41]. The organization environment includes those attributes within an organization that are supportive of using technology; they include leadership commitment to use technology, availability of resources to acquire it, and structural readiness for its deployment [42]. The external environment contains those factors outside the organization that may either encourage or discourage the development of innovations through regulations and laws at local, national and international levels, competitive nature of business and industry, professional associations and networks etc. By bringing all of these three dimensions together, TOE creates a structured way for researchers to study technology adoption by organizations [43].

Diffusion of Innovation theory complements this framework by explaining how the perceived attributes of an innovation influence its adoption. According to this perspective, innovations that demonstrate clear advantages compared with existing practices are more likely to be adopted by organizations and individuals [44]. When stakeholders perceive a technology as offering improved efficiency, enhanced collaboration, or superior performance outcomes, their willingness to adopt the innovation increases significantly [45]. In the context of BIM supported green building development, the perceived benefits of BIM technologies play an important role in shaping organizational decisions regarding technology adoption [46].

The diffusion of innovation (DOI) model is complementary to the technology acceptance model, as it provides insight into the extent to which the perception of an innovation’s characteristics influences its adoption. The DoI model suggests that when an organization perceives a new technology as providing advantages over the current process, they will be more likely to accept the technology for use within their organization [44]. Similarly, when an individual believes a technology will allow them to complete tasks at greater efficiency, collaborate better, or provide better results than alternative options, he/she will have a higher likelihood of adopting that technology [45]. Perceived benefits associated with BIM technologies will greatly impact an organization’s decision-making process relative to accepting or rejecting the use of those technologies in supporting green building design using BIM [46].

This study is based on a combined approach of technology-orientation (e.g., technological competence, top-management-support) and organization-orientation (e.g., organizational-readiness, external-support) in order to understand which factors are influential for the integration of BIM-supported green building-practices into construction companies. The variables examined by this research include technological competence, top-management-support, organizational-readiness, external-support, environmental-sustainability-orientation, as well as the perceived relative advantage, all of which together provide an insight into the interplay of organizational-environmental-technological factors that determine the adoption of digital innovations in sustainable-construction contexts.

### Technological competence and BIM supported green building adoption

Technological competence is about having the necessary technology (infrastructure, skills and knowledge) to be able to use new technologies such as BIM. In order for an organization to successfully adopt BIM they need to have a high level of technological capability which includes, appropriate software systems, information management systems and people with the appropriate training to operate BIM tools [47]. If an organization does not have the required technological competences then they will find it difficult to achieve BIM adoption due to its high degree of technical complexity and the number of additional resources needed [48].

Beyond possessing tools and infrastructure, technological competence also determines how effectively an organization can exploit BIM for sustainability-specific functions such as energy simulation, embodied-carbon estimation, and lifecycle performance analysis. Firms with mature digital capabilities can integrate these analytical modules into existing workflows with lower switching costs, whereas firms with limited competence tend to confine BIM to basic modeling and coordination, leaving its green-building potential underutilized. Technological competence is therefore not merely a precondition for adoption but a factor that shapes the depth and sustainability value of the adoption itself [49].

- **H1: Technological competence positively associated BIM supported green building adoption.**

### Top management support and BIM supported green building adoption

Top management support is the level of commitment that senior leadership has for advancing technology innovation throughout an organization. Typically, in order for companies in the construction industry to adopt BIM, they are required to invest large sums of money to fund the new processes, and they will need to restructure their organizations and provide employees with necessary training in order to effectively implement this new process [50]. Therefore, it is imperative that there be a high level of commitment from senior leadership (top management) as they have the authority to allocate resources to enable the strategic objectives to be accomplished and to encourage employees to undergo the needed change [32].

Leadership commitment has greater implications for sustainability-oriented innovation because sustainable building practices usually require an organization to embrace novel design methods, invest in more sophisticated technology and engage with many different stakeholders [51]. When the upper levels of management provide active support for BIM implementation, staff will be more inclined to view BIM adoption as a high priority strategic organizational objective versus simply a voluntary technological alternative. In addition, when leaders support the adoption of BIM, they encourage the establishment of organizational policies and training programs that help ensure that BIM technology can be effectively implemented by their organization [36].

As a result, organizations with strong managerial support are more capable of integrating BIM into their construction processes and promoting its use for sustainable building development.

- **H2: Top management support positively associated BIM supported green building adoption.**

### Organizational readiness and BIM supported green building adoption

Organisational readiness is the degree to which organisations have developed the requisite capacity, in terms of their processes, people, etc to enable the implementation of new technologies [52]. To implement a Building Information Modelling (BIM) system within an organisation; it will be required to set up suitable training programs for staff, to develop and implement digitally enabled workflows that facilitate collaboration and information sharing amongst all relevant stakeholders involved in projects [53].

Organizations with a high level of readiness for BIM adoption have the structure and personnel in place to facilitate their transition to a digital construction environment. This means that organizational readiness encompasses employee’s willingness to use new technology as well as an organization’s ability to modify its current workflow to accommodate changes resulting from technological innovations [54].

Organizational readiness is especially significant in the field of green building development since it will require collaboration among architects, engineers and project managers using BIM to support a sustainability analysis through all stages of the design and construction process [42]. Thus, organizations which have effectively transitioned from paper-based to digitally enabled processes are better equipped to utilize BIM in their application of sustainable construction practices.

- **H3: Organizational readiness positively associated BIM supported green building adoption.**

### External support and BIM supported green building adoption

External support is defined as how external influences from institutions, government policy initiatives, or networks (industry) in an organization’s environment affect its ability to accept technological advancements [55]. In most countries, government agencies and other professional associations have implemented policies that either regulate BIM use by providing some type of incentive for BIM use or education/training. As such, they provide a framework to lower the uncertainty that technology can create, allowing construction companies to be encouraged to embrace new technologies [56].

External support also includes collaboration with industry partners and technology providers as well as research institutions that facilitate the diffusion of BIM technologies across the construction sector. Such collaborations enable knowledge sharing, technical guidance and access to specialized resources to facilitate the implementation of BIM [57].

Within the context of sustainable construction, regulatory frameworks and environmental policies can further motivate organizations to adopt BIM technologies because digital modeling tools enable more accurate environmental assessments and sustainability reporting [54]. Consequently, organizations operating in environments with strong institutional support for digital innovation and sustainability are more likely to adopt BIM supported green building practices.

- **H4: External support positively associated BIM supported green building adoption.**

### Environmental sustainability orientation and BIM supported green building adoption

The amount to which an organization considers its environmental responsibilities when making strategic decisions and conducting daily operations is referred to as the environmental sustainability orientation [58]. An organization that has a high level of commitment to sustainability will be much more inclined to implement new technology such as BIM to facilitate environmentally sustainable construction processes [59].

Construction organizations have the opportunity to use BIM technologies to assist them in measuring and assessing the environmental performance of their building designs and constructions [60]. As such, these organizations can evaluate the efficiency of energy usage, assess the materials used in constructing buildings, and ensure optimal building operation to achieve minimal negative impacts on the environment. Therefore, it follows logically that organizations with an emphasis on sustainability would be more interested in using BIM since they understand that digital models can help support the achievement of their own environmental goals [61].

In addition, with the rising awareness among the general public about the environment and an increasingly higher expectation from stakeholders for sustainable buildings, construction companies are beginning to consider how they can incorporate sustainability in to their project management processes [62]. Using BIM for green building projects is one way to achieve this goal, as it allows for data-driven sustainability assessments and life cycle performance evaluations

- **H5: Environmental sustainability orientation positively associated BIM supported green building adoption.**

### Relative advantage and BIM supported green building adoption

Relative advantage is when a new practice provides better results than what was being done before. The theory on diffusion of innovations suggests that the advantages provided in terms of performance will have a greater likelihood of being implemented in organizational settings [61]. For example, some of the possible ways BIM can help with the process of building projects, such as better coordinated projects, increased precision for designs, fewer construction mistakes and more efficiency in managing projects, all represent potential advantages that could lead to adoption [56].

BIM provides numerous environmental benefits with respect to green building development through enhancing the ability for conducting advanced sustainability analysis and simulating the lifecycle performance of buildings [62]. By employing these capabilities, organizations can provide environmentally effective design options, optimize building performance, and minimize resource usage during all phases of the construction lifecycle [42].As such, when construction professionals feel that BIM has significantly enhanced their operations and environmentally positive contributions; they are more likely to adopt BIM technology [55]. The perceived relative advantages of BIM is one of the most critical factors influencing whether or not an organization will incorporate BIM into their sustainable construction strategy.

- **H6: Relative advantage positively asscoiated BIM supported green building adoption.**

### BIM-supported green building adoption and smart and sustainable city development: contextual background

Smart cities focus on developing sustainable urban environments through integrating digital technology into physical (environmentally-friendly) infrastructures in order to increase the sustainability and efficiency of urban areas [61]. Sustainable buildings are a key part of an urban ecosystem due to their contributions towards reducing energy consumption, enhancing environmental performance and managing resources efficiently [52].

Adoption of green building utilizing BIM will be able to provide significant support toward smart city development through supporting digital solutions for collecting and managing building information at all stages of urban infrastructure asset life cycles [53]. Utilizing BIM enables real-time assessment of building performance; supports data-driven decisions; and facilitates the implementation of smart building technologies to optimize operational efficiencies [63].

As urban sustainability is pursued through green building strategies within cities; as such, increasing use of BIM-supported green building methods will become increasingly important in assisting digital infrastructure management and enhancing environmental performance of urban systems.

## Methodology

**Fig 1:**
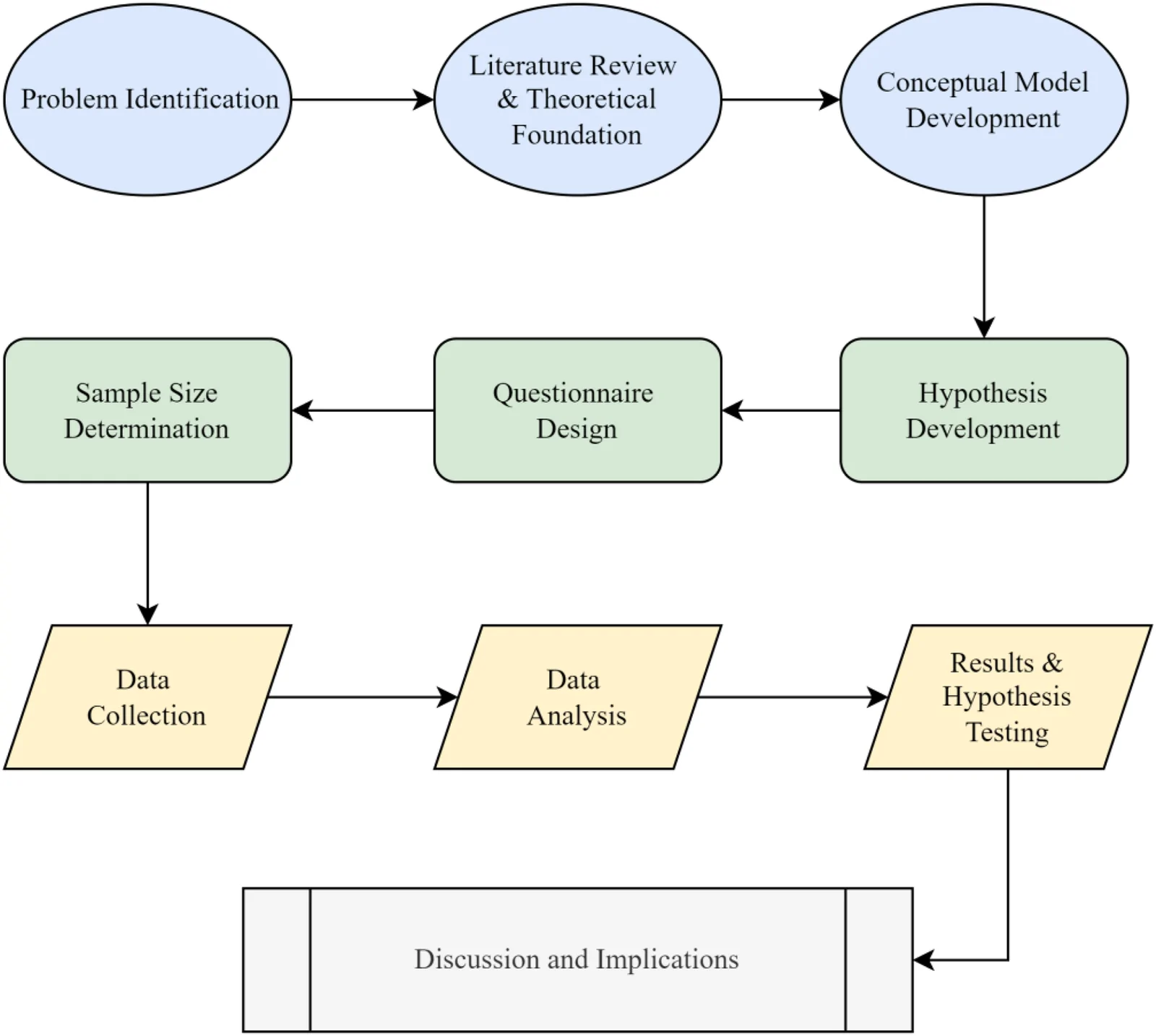
Flow chart of the study.

This study employs a quantitative design for identifying the determinants of BIM supported green building adoption by construction organizations and how this contributes to Smart City/Sustainable City Development (Fig 1). Quantitative methods have been frequently used in Technology Adoption Studies as they allow researchers to systematically analyze relationships among numerous constructs utilizing various statistical models. The research will collect structured survey data from construction professionals and utilize Structural Equation Modeling (SEM) to analyze the relationships among the technological, organizational, and environmental determinants of BIM supported Green Building Adoption. This research design will allow the researcher to assess both the measurement properties of the constructs being analyzed as well as the structural relationship among the constructs through an integrated analytical process.

This study’s conceptual framework utilizes Technology Organization Environment (TOE) framework as well as Diffusion of Innovation (DoI) theory for understanding factors that are representative of technological competencies, organizational capabilities, environmental influences, and perceived technological benefits associated with Building Information Model (BIM) adoption. Using these two theories as a basis for its conceptual framework, this study developed a structural model which will assess the relationships among technological competencies, top management support, organizational readiness, external support, environmental sustainability orientation, and perceived relative advantages, with BIM-supported green building adoption, as well as, assess the contributions of BIM-supported green building adoption toward smart and sustainable city development.

### Population and sampling

The subject population for this study consists of those individuals who are employed in a professional capacity within the construction industry of Saudi Arabia and have experience with the use of digital construction technologies, sustainable building methods, or project management techniques. This would encompass Architects, Engineers, Project Managers, BIM Coordinators, Consultants, and other Construction Stakeholders that have direct involvement in the Planning, Design, or Implementation phases of a construction project. By selecting such respondents for this study, it will ensure that the opinions expressed reflect an educated perspective on BIM implementation and sustainable construction practices.

A structured survey method was applied to gather information from the construction community through an array of organizations; selection of participants involved a purposeful sampling approach that focused on selecting participants as being appropriate to the study due to their applicable experience within the construction industry and applicable knowledge of BIM systems and/or green building practices. Purposeful sampling is frequently utilized in studies of technology adoption as this methodology enables researchers to access participants whose skills are applicable to providing meaningful responses in relation to the conceptual frameworks of the research. Purposeful sampling nonetheless carries recognized limitations: because participants were not selected through random probability methods, the sample may not be fully representative of the broader population of Saudi construction professionals, and those more interested in BIM and sustainability may have been more likely to participate, raising the risk of non-response bias. These risks were partly mitigated by using multiple independent recruitment channels rather than a single network (Data Collection Procedure) and applying consistent eligibility criteria across all channels, which helped broaden the demographic spread of the final sample. Even so, the findings should be interpreted as reflecting professionals accessible through these channels rather than a strictly representative cross-section of the industry.

In order to establish the optimal sample size for this study, we employed two well-established methodologies to be certain of the adequacy of the sample size for the purpose of ensuring there would be enough statistical power and for the sample size to be representative of the population being studied. Initially, the sample size was determined through a priori power analysis for multiple regression in the G*Power software program. In this instance, we applied a significance level (alpha) of 0.05, a statistical power of 0.95, and six predictor variables, all of which corresponded to the independent constructs within the structural model. This provided a minimum sample size of 146 participants necessary to have a reliable measure of detecting a medium sized effect. Next, the sample size was determined using Cochran’s Formula, a method commonly utilized in the context of survey-based research to identify the appropriate sample size for large populations [2,64,65]. The Cochran’s Formula was employed at a 95% confidence interval and 5% margin of error, and identified a minimum sample size of 384 participants as necessary. Ultimately, our final dataset of 389 valid responses exceeded the recommendations of both methodologies; therefore, it can be concluded that our sample size is sufficiently large for conducting robust statistical analyses and structural equation modeling.

### Questionnaire development

The questionnaire consisted of two sections. The first section collected demographic and professional information, including gender, age, educational qualification, professional experience, organizational position, organization type and size, BIM experience, and involvement in green building projects. The second section measured the seven constructs included in the conceptual model: technological competence, top management support, organizational readiness, external support, environmental sustainability orientation, relative advantage, and BIM-supported green building adoption.

The initial measurement instrument comprised 35 items, with five items assigned to each construct. The items were adapted from established technology-adoption, BIM-adoption, and corporate environmental-orientation measures rather than developed without a theoretical basis. Technological competence, top management support, organizational readiness, and external support were informed by established Technology–Organization–Environment instruments and previous organizational BIM-adoption studies. Relative advantage was adapted from established innovation-adoption measures, while environmental sustainability orientation was based on measures of corporate environmental orientation. BIM-supported green building adoption was operationalized using organizational BIM-adoption measures contextualized specifically for sustainable and green building applications.

The original measures were adapted to the present research context through a structured contextualization process. Generic references to “information technology,” “innovation,” or “technology adoption” were replaced with references to BIM implementation. Where conceptually appropriate, references to sustainable construction, green building development, environmental analysis, and lifecycle performance were incorporated. The adaptations retained the conceptual meaning of the original measures while making the wording understandable and relevant to professionals working in architecture, engineering, and construction organizations.

Content and face validity were assessed by a panel of five experts consisting of two university academics specializing in BIM and sustainable construction, two BIM managers with industry experience, and one green building and sustainability consultant. The experts independently evaluated each item for relevance, clarity, representativeness, and suitability for the Saudi construction context. The item-level content validity indices ranged from 0.80 to 1.00, while the scale-level content validity index based on the average method was 0.94, indicating satisfactory content validity. Based on the experts’ recommendations, minor wording changes were made to remove ambiguity, simplify technical terminology, and ensure that all statements were expressed at the organizational level.

A pilot survey was subsequently administered to 30 construction professionals with BIM or sustainable-construction experience. These respondents were not included in the final sample. Participants were asked to assess the clarity, comprehensibility, completion time, and relevance of the questionnaire. The pilot reliability coefficients ranged from 0.76 to 0.89 across the seven constructs, exceeding the minimum value of 0.70. No item required deletion at the pilot stage, although minor grammatical and contextual modifications were made before distributing the final questionnaire.

All construct items were measured using a five-point Likert scale ranging from 1, “strongly disagree,” to 5, “strongly agree.” The complete questionnaire, together with the adaptation sources for each construct, is presented in Appendix B.

This revision corrects the manuscript’s present unsupported description of “eight constructs”; the model actually measures six predictors and one endogenous adoption construct. The smart-and-sustainable-city relationship is discussed contextually rather than tested as a separate latent construct.

### Data collection procedure

The electronic questionnaire presented in Appendices A and B was distributed to architectural, engineering, and construction professionals working in Saudi Arabia. Respondents were recruited through multiple channels to reduce dependence on a single source. These channels included direct email and LinkedIn outreach to architects, civil engineers, project managers, BIM managers and coordinators, consultants, and other construction professionals; circulation through professional networks and online forums concerned with BIM, construction management, green buildings, and sustainable construction in Saudi Arabia; and internal forwarding of the survey within contractor, consultancy, developer, and government organizations.

The survey remained accessible for eight weeks during the data-collection period in early 2026. Potential participants received a short explanation of the research objectives, eligibility requirements, voluntary nature of participation, and confidentiality of their responses before accessing the questionnaire. To be eligible, respondents were required to be currently employed in the Saudi construction industry and to possess professional knowledge or experience related to construction project delivery, BIM, digital construction technologies, or sustainable building practices alinged with previous research [66].

Because the survey link was distributed through professional groups and could be forwarded internally by organizational contacts, the exact number of individuals who received or viewed the invitation could not be reliably determined. Consequently, a conventional response rate could not be calculated. This limitation is acknowledged because professionals who were more interested in BIM and sustainable construction may have been more likely to participate. After data screening, 389 complete and usable responses were retained for the final analysis.

### Data analysis technique

Collected data were analyzed using partial least squares structural equation modeling (PLS-SEM) via SmartPLS software. PLS-SEM was selected over covariance-based SEM (CB-SEM) for three reasons consistent with established methodological guidance [67,68]. First, this study’s objective is primarily predictive and exploratory identifying which determinants most strongly explain variance in BIM-supported green building adoption which aligns with PLS-SEM’s prediction-oriented approach, whereas CB-SEM is better suited to confirmatory theory testing of an already well-established model. Second, the structural model combines constructs from two distinct theoretical traditions (TOE and DOI) that have not previously been integrated in this way, and PLS-SEM accommodates such theoretically novel, less-established models more robustly than CB-SEM, which assumes a precisely specified and previously validated model structure. Third, PLS-SEM does not require multivariate normality and performs reliably with the moderate-to-large sample size and reflective multi-item measurement approach used in this study. For these reasons, PLS-SEM was judged more appropriate than CB-SEM for the exploratory, prediction-oriented aims of this research.

A two-stage process was applied to conduct the analysis; first the measurement model was evaluated and then the structural model was evaluated. In the first stage, the measurement model evaluation was conducted to evaluate the reliability and validity of the constructs and this was done by assessing indicator loadings, internal consistency reliability (composite reliability and Cronbach’s alpha), convergent validity (average variance extracted), and discriminant validity (relationships among constructs) [69]. Indicators’ reliability was assessed by determining if the factors loading on each measurement item indicated high reliability. Internal consistency reliability was determined by calculating both composite reliability and Cronbach alpha values to determine if all indicators measuring a construct indicated sufficient inter-item correlation to demonstrate internal consistency reliability. Convergent validity was assessed by determining the average variance extracted (AVE) value for each construct to determine if the shared variance between each indicator measuring a construct demonstrated adequate convergence [70].

Finally, discriminant validity was assessed to ensure that each construct was empirically distinct from other constructs included in the model. Discriminant validity was assessed based on the relationships among constructs as compared to standard discriminant validity criteria. When reliability and validity are established for the measurement model, the constructs are adequately measured by their respective indicators.

Following confirmation of the measurement model, the structural model was evaluated to assess the hypothesized relationships among the constructs. The structural model analysis evaluated the magnitude and statistical significance of the relationship between independent and dependent variables as indicated by the path coefficients, t-values, and statistical significance values obtained through bootstrapping methods. Finally, the explanatory power of the model was assessed utilizing coefficient of determination values to indicate the proportion of variance accounted for in the dependent constructs.

This study was reviewed and approved by the Faculty of Engineering and Information Technology Board, Taiz University (approval reference no. 110–26EIT-79B). All procedures involving human participants complied with the ethical standards of the institutional research committee. Participation was entirely voluntary and anonymous. Before beginning the questionnaire, all respondents were presented with an information statement describing the purpose of the study, the voluntary nature of participation, their right to withdraw at any point without consequence, and assurances that responses would remain confidential and be used solely for academic purposes. Informed consent was obtained from all participants, who provided consent by proceeding to complete the survey after reading this statement. No personally identifiable information was collected, and all data were anonymized prior to analysis. No minors were involved in this study.

### Findings

This section presented the empirical findings of the study by evaluating the measurement and structural models. The measurement model assessment confirmed satisfactory reliability, convergent validity, and discriminant validity of all constructs through reliability coefficients, AVE values, HTMT ratios, Fornell–Larcker criterion, and cross loading analysis. The structural model results further examined the hypothesized relationships among the determinants of BIM supported green building adoption. Bootstrapping analysis demonstrated that all proposed hypotheses were statistically significant and positively related to the dependent variable. Overall, the findings highlight that technological competence, top management support, and relative advantage are the most influential drivers of BIM supported green building adoption, while environmental sustainability orientation, external support, and organizational readiness provide additional supporting effects.

### Demographics

The demographic results in Table 1 indicate that the sample primarily consists of experienced construction professionals with relevant exposure to BIM and sustainable construction practices. Prior to analysis, the dataset was screened for missing values. The only missing responses occurred in a small number of optional demographic items (ranging from 1.3% to 2.8% per category), where some respondents chose not to disclose particular background information. Because these demographic variables were used solely to describe the sample and were not included as indicators in the structural model, the missing demographic values had no effect on the PLS-SEM estimation. All responses to the substantive construct items used for model estimation were complete, and the final dataset of 389 valid responses therefore contained full data for every measurement item entered into the analysis.

**Table 1:** Respondents details.

Variable	Category	Frequency	Percentage (%)
**Gender**	Male	282	73.4
	Female	101	26.3
	Missing	6	1.5
**Age Group**	20–29 years	96	24.7
	30–39 years	158	40.6
	40–49 years	87	22.4
	50 years and above	38	9.8
	Missing	10	2.6
**Education Level**	Diploma	72	18.5
	Bachelor’s degree	203	52.2
	Master’s degree	89	22.9
	PhD	18	4.6
	Missing	7	1.8
**Work Experience**	Less than 5 years	104	26.7
	5–10 years	148	38.0
	11–15 years	83	21.3
	More than 15 years	47	12.1
	Missing	7	1.8
**Position**	Project Engineer	118	30.3
	Site Engineer	74	19.0
	Architect	52	13.4
	BIM Manager/Coordinator	36	9.3
	Project Manager	91	23.4
	Other	11	2.8
	Missing	7	1.8
**Organization Type**	Contractor	173	44.5
	Consultant	89	22.9
	Developer	61	15.7
	Government Agency	51	13.1
	Other	10	2.6
	Missing	5	1.3
**Organization Size**	Small (1–50 employees)	94	24.2
	Medium (51–250 employees)	166	42.7
	Large (251 + employees)	121	31.1
	Missing	8	2.1
**BIM Experience**	No experience	41	10.5
	Less than 2 years	106	27.2
	2–5 years	158	40.6
	More than 5 years	73	18.8
	Missing	11	2.8
**Green Building Project Experience**	None	68	17.5
	1–2 projects	121	31.1
	3–5 projects	129	33.2
	More than 5 projects	60	15.4
	Missing	11	2.8

The majority of respondents were male (73.4 percent), reflecting the traditional gender distribution within the construction industry. Most participants were between 30 and 39 years old (40.6 percent), suggesting that the dataset largely represents mid-career professionals actively engaged in industry practices. In terms of education, more than half of the respondents held a bachelor degree (52.2 percent), indicating a strong professional educational background. The largest proportion of respondents had five to ten years of work experience (38 percent), which implies sufficient industry exposure to digital construction technologies. Project engineers and project managers formed the largest professional groups, highlighting the practical involvement of respondents in construction project execution. Contractors represented the largest organizational category (44.5 percent), while most respondents worked in medium sized organizations (42.7 percent). Notably, a significant proportion of respondents reported two to five years of BIM experience (40.6 percent) and involvement in multiple green building projects, indicating that the sample possesses relevant practical experience to provide informed insights into BIM supported green building adoption.

Although a minority of respondents reported no direct BIM experience (10.5%) or no prior green building project involvement (17.5%), these participants were retained because the study measures organizational-level adoption determinants and perceptions rather than individual BIM proficiency; professionals within adopting organizations can meaningfully report on their firm’s technological competence, leadership support, and readiness regardless of their own hands-on BIM use. Their inclusion also captures the perspective of professionals at earlier stages of the adoption curve, which is relevant to understanding adoption determinants across the diffusion spectrum.

### Common method bias assessment

Because all constructs were measured using a single self-reported questionnaire administered to the same respondents at one point in time, the potential for common method bias (CMB) was assessed. First, Harman’s single-factor test was conducted, and the first unrotated factor accounted for 38.72% of the total variance. This value is below the recommended threshold of 50%, indicating that no single factor dominated the total variance. Second, following Kock (2015) [71], a full collinearity assessment was performed by examining the variance inflation factor (VIF) values obtained from the PLS-SEM analysis. All full-collinearity VIF values were below the conservative threshold of 3.3, ranging from 1.214 to 2.876, confirming the absence of serious common method bias. Taken together, these findings indicate that common method bias was unlikely to have materially influenced the study results.

### Convergent validity and reliability analysis

Table 2 results based on the measurement model indicate high levels of reliability and validation evidence (convergent) for all studied variables. Reliability is demonstrated by Cronbach alpha coefficients ranging from 0.716 to 0.873, as all were greater than the generally accepted 0.70 threshold; therefore, there appears to be an appropriate amount of internal consistency among the measurement items. Additionally, both rho a and rho c composite reliabilities were higher than the suggested 0.70 threshold, demonstrating that the studied variables represent reliable representations of the theoretically-derived variables. The Average Variance Extracted (AVE) ranges from 0.638 to 0.799 for all variables, each of which was significantly greater than the minimum threshold of 0.50; therefore, each variable explained a large portion of variance associated with each measure. Specifically, organizational readiness and technological competence were found to have particularly strong evidence of convergent validity (AVEs = 0.799 and 0.791) this suggests that measures for these variables provide adequate representation. Relative advantage exhibited the lowest Cronbach’s Alpha coefficient (0.716), however it is still considered to be within an acceptable reliability limit for additional structural analysis. As such, the results demonstrate robust support for the reliability and validity of the measurement model; therefore, the measured variables can be used appropriately for subsequent structural equation modeling analyses.

**Table 2:** Convergent validity and reliability.

Constructs	Cronbach’s alpha	Composite reliability (rho-a)	Composite reliability (rho-c)	Average variance extracted (AVE)
**Environmental Sustainability Orientation**	0.815	0.821	0.891	0.732
**External Support**	0.814	0.832	0.879	0.648
**Organizational Readiness**	0.873	0.875	0.922	0.799
**Relative Advantage**	0.716	0.717	0.841	0.638
**Technological Competence**	0.860	0.894	0.918	0.791
**Top Management Support**	0.835	0.839	0.901	0.753

Item-level assessment was conducted prior to evaluating construct-level reliability and validity. Of the five items originally developed for each of the six determinant constructs, between three and four items were retained per construct following measurement model purification, based on outer loading thresholds and their effect on internal consistency reliability and convergent validity. Retained item loadings ranged from 0.669 to 0.965. Consistent with established PLS-SEM guidelines indicators with loadings between 0.40 and 0.70 are considered for removal only when their deletion increases internal consistency reliability or convergent validity above the recommended threshold [72]. The single retained item below 0.70 (External Support, item 3 = 0.669) was evaluated on this basis: its removal did not improve composite reliability or AVE beyond the recommended thresholds (CR = 0.879, AVE = 0.648 with the item included), so it was retained. All remaining retained items exceeded 0.70, with the majority above 0.80, confirming adequate indicator reliability across the measurement model

### Discriminant validity analysis

The discriminant validity of the constructs was assessed using the Heterotrait–Monotrait ratio of correlations (HTMT) as shown in Table 3. The results indicate that all HTMT values are well below the recommended threshold of 0.85, demonstrating adequate discriminant validity among the constructs. The highest HTMT value observed in the model is between top management support and relative advantage (0.556), which remains significantly lower than the critical threshold, suggesting that these constructs, although conceptually related, are empirically distinct. Similarly, the relationships between technological competence and organizational readiness (0.310) as well as technological competence and relative advantage (0.323) indicate moderate associations without compromising construct independence. The remaining HTMT values are relatively low, ranging from 0.112 to 0.342, further confirming that the constructs measure distinct theoretical concepts within the model.

**Table 3:** HTMT analysis.

Constructs	Environmental Sustainability Orientation	External Support	Organizational Readiness	Relative Advantage	Technological Competence	Top Management Support
**Environmental Sustainability Orientation**						
**External Support**	0.115					
**Organizational Readiness**	0.117	0.112				
**Relative Advantage**	0.217	0.197	0.277			
**Technological Competence**	0.148	0.191	0.310	0.323		
**Top Management Support**	0.225	0.206	0.342	0.556	0.494	

The Fornell–Larcker criterion shown in Table 4 was applied to evaluate further discriminant validity of constructs of measurement model. Results indicate that the square root of the average variance extracted for each construct (diagonal) exceeds all of the inter-construct correlation in same row and column. Environmental sustainability orientation has an AVE square root 0.855. This is larger than the corresponding correlations with external support (0.061); organizational readiness (0.095); relative advantage (0.152); technological competence (0.085); and top management support (0.184). In addition, similarly, technological competence (0.889); organizational readiness (0.894); and top management support (0.868) have diagonal values larger than the respective correlations with all other constructs in the model. Overall, these results support that each construct shares more variance among its own measures than with other constructs in the measurement model. Therefore, based on these findings we can conclude there are sufficient supports for adequate discriminant validity in the measurement model therefore, the constructs represent different conceptual dimensions as part of the structural model.

**Table 4:** Fornell–Larcker analysis.

Constructs	Environmental Sustainability Orientation	External Support	Organizational Readiness	Relative Advantage	Technological Competence	Top Management Support
**Environmental Sustainability Orientation**	0.855					
**External Support**	0.061	0.805				
**Organizational Readiness**	0.095	−0.097	0.894			
**Relative Advantage**	0.152	0.103	0.220	0.799		
**Technological Competence**	0.085	0.160	0.266	0.258	0.889	
**Top Management Support**	0.184	0.161	0.283	0.428	0.420	0.868

The cross-loading analysis in Table 5 was conducted to further examine discriminant validity by comparing the loading of each indicator on its corresponding construct with its loadings on other constructs. The results confirm that all measures have a larger factor (loading) for the appropriate construct than they do for the other constructs in the model. For example, three of the measures related to environmental sustainability orientation are extremely large at 0.909, 0.795 & 0.858 relative to their corresponding loadings (0.111 & 0.104) on other constructs. Similarly, there are measures of external support with very large loadings (0.669–0.895) relative to their corresponding loadings on other constructs. Also, organizational readiness has extremely large loadings (0.855–0.914). High factor loadings (0.778–0.816) were found in relative advantage. High factor loadings (0.960 & 0.965) were also identified in technological competence. Top management support also had significant factors (0.834–0.914). More importantly, the cross-loading values of each measure across non-associated constructs were significantly smaller than the primary factor loadings; this supports that each measure was significantly stronger associated with its intended construct than it was with the non-associate constructs. The above results will be used as additional evidence that discriminant validity has been demonstrated within the measurement model.

**Table 5:** Cross-loading analysis.

Variables	Environmental Sustainability Orientation	External Support	Top Management Support	Organizational Readiness	Relative Advantage	Technological Competence
**EO-TOE 1**	0.909	0.021	0.189	0.058	0.124	0.107
**EO-TOE 3**	0.795	0.114	0.230	0.181	0.250	0.142
**EO-TOE 4**	0.858	0.028	0.057	0.013	0.026	−0.027
**ES-TOE 1**	0.071	0.871	0.098	−0.106	0.052	0.066
**ES-TOE 2**	−0.045	0.669	0.145	−0.023	−0.092	0.086
**ES-TOE 4**	0.099	0.895	0.174	−0.088	0.193	0.182
**ES-TOE 5**	0.050	0.764	0.103	−0.086	0.142	0.177
**MS-TOE 2**	0.174	0.226	0.834	0.351	0.406	0.379
**MS-TOE 3**	0.221	0.151	0.914	0.274	0.369	0.374
**MS-TOE 5**	0.082	0.045	0.854	0.114	0.341	0.342
**OR-TOE 1**	0.093	−0.054	0.122	0.855	0.176	0.243
**OR-TOE 2**	0.072	−0.115	0.304	0.910	0.211	0.218
**OR-TOE 3**	0.090	−0.090	0.325	0.914	0.201	0.252
**RA-DOI 1**	0.102	0.078	0.344	0.193	0.816	0.294
**RA-DOI 2**	0.110	0.106	0.426	0.144	0.778	0.113
**RA-DOI 5**	0.152	0.062	0.258	0.188	0.801	0.206
**S8TC-TOE 4.**	0.126	0.129	0.290	0.231	0.167	0.722
**TC-TOE 1**	0.055	0.136	0.411	0.240	0.265	0.960
**TC-TOE 3**	0.060	0.161	0.408	0.241	0.245	0.965

### Structural model analysis

**Fig 2:**
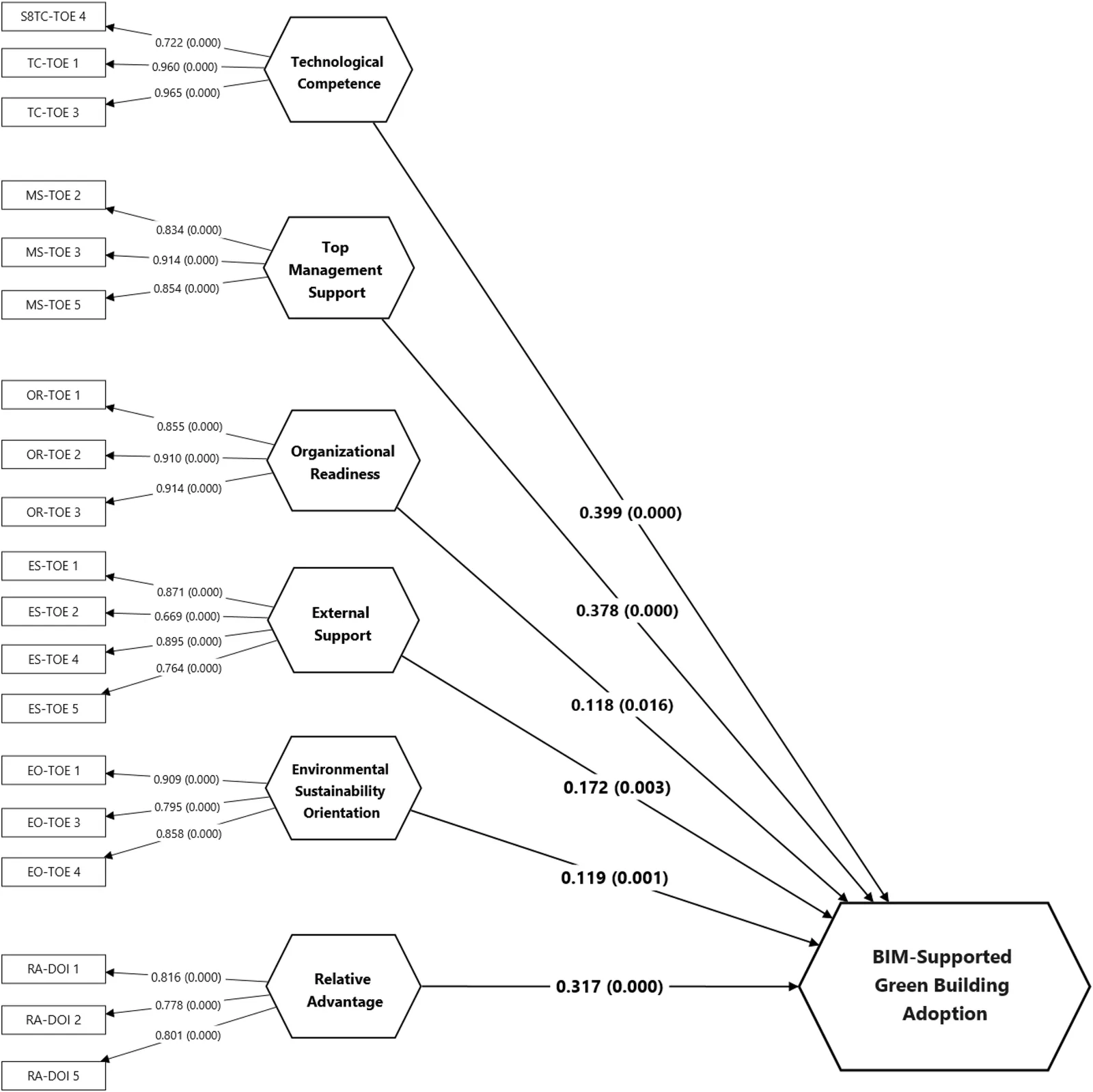
Structural model with path loadings and P values.

The structural model depicted in Fig 2 illustrates the relationships between the six determinants and BIM-supported green building adoption. It was found that technological competence has the most profound influence on the use of BIM for sustainable construction practices (β = 0.399; p < 0.001) due to the fact that those with higher levels of digital capability and knowledge will be able to develop an organization that is capable of implementing BIM based sustainable construction practices. Similarly, top management support was also shown to have a very large positive effect (β = 0.378; p < 0.001). This indicates that if top management is committed to supporting the implementation of BIM into their organization’s construction processes, this will help facilitate their organization’s adoption of BIM technologies. Additionally, it was demonstrated that relative advantage has a moderate to high positive impact (β = 0.317; p < 0.001); this implies that when organizations see BIM as providing them with tangible sustainability and/or operational advantages over other alternatives they are more willing to implement the technology. External support showed a small but statistically significant positive association with BIM-supported green building adoption (β = 0.172; p = 0.003), suggesting that regulatory encouragement and industry support provide some additional influence on an organization’s decision to implement BIM-supported sustainable construction practices. Environmental sustainability orientation was also positively and significantly related to adoption (β = 0.119; p = 0.001), reflecting that organizations prioritizing environmental responsibility are more inclined to incorporate BIM into their construction practices. Finally, organizational readiness showed a positive and significant, though comparatively smaller, association (β = 0.118; p = 0.016), indicating that firms with appropriate internal structures and prepared workflows are better positioned to adopt BIM.

**Fig 3:**
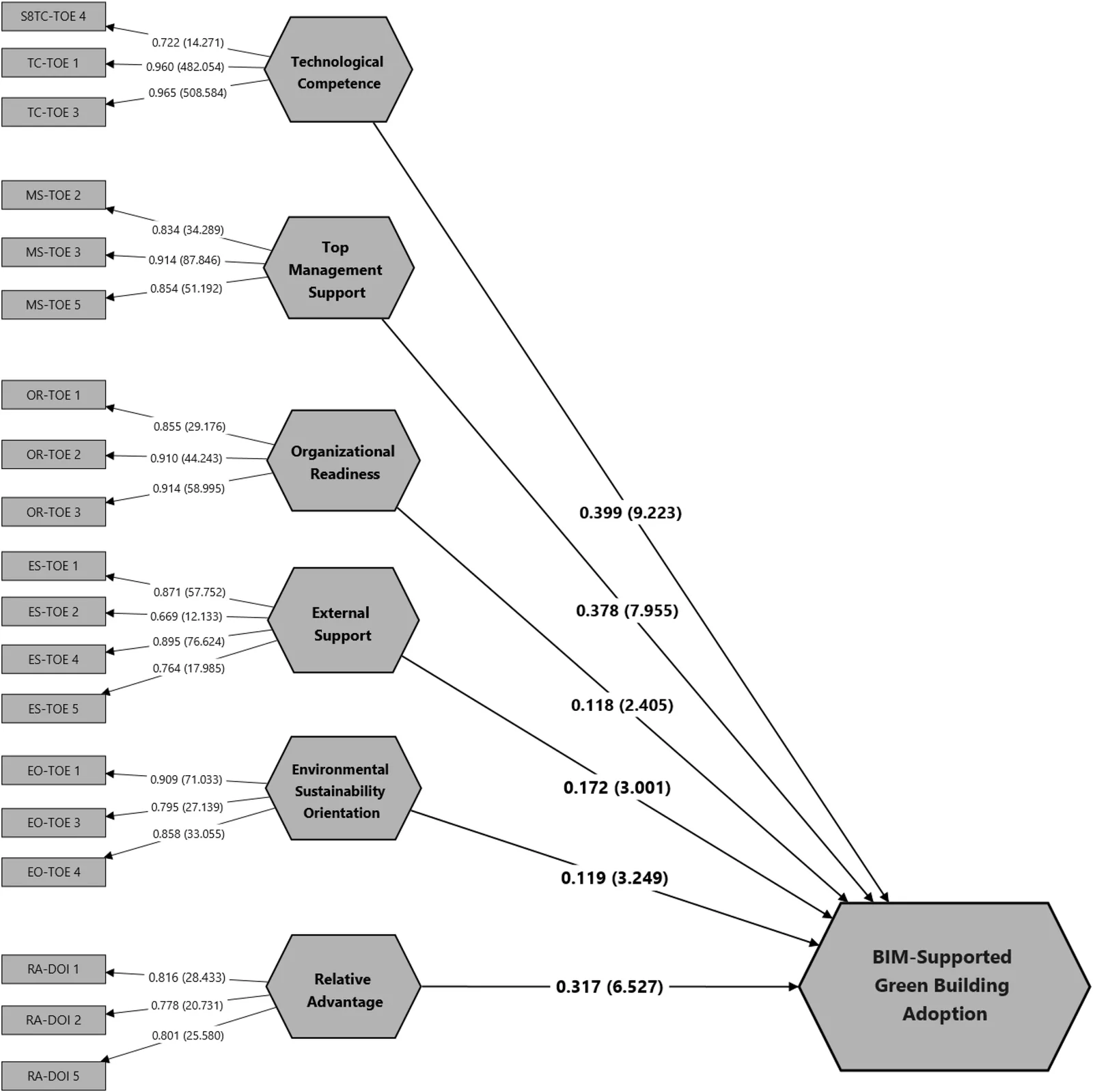
Model with path loadings and T values.

The data shown in Fig 3 indicates that the hypothesised relationships all have a positive and statistically significant relationship to the extent of BIM-supported green building adoption. In particular, technological competency has by far the largest relationship (β = 0.399; t = 9.223); as such organisations with greater technical capabilities will be more likely to apply BIM within their sustainable construction practices. As such, top management support is also found to have a considerable positive relationship to the adoption of BIM (β = 0.378; t = 7.955); this again supports the notion that for technology to be adopted, organisational leadership needs to provide a commitment to it. The relative advantages of using BIM were also found to positively affect its use (β = 0.317; t = 6.527). This suggests that if an organisation perceives that BIM would offer them additional benefits, they may therefore adopt it. Finally, external support was found to have a positive relationship with BIM adoption (β = 0.172; t = 3.001), as did environmental sustainability orientation (β = 0.119; t = 3.249) and organizational readiness (β = 0.118; t = 2.405). This suggests that both how much organisations focus upon their own sustainability and the level of institutional support received by organisations can positively affect the adoption of BIM.

### Structural-model outputs: R², adjusted R², f², Q², SRMR

The explanatory and predictive quality of the structural model was assessed. The model explained a substantial proportion of the variance in BIM-supported green building adoption, with an R² value of 0.684 and an adjusted R² value of 0.676, indicating substantial explanatory power. Predictive relevance was confirmed through the blindfolding procedure, which produced a Q² value of 0.451, exceeding zero and demonstrating satisfactory predictive relevance for the endogenous construct. Furthermore, the standardized root mean square residual (SRMR) was 0.062, which is below the recommended threshold of 0.08, indicating an acceptable model fit. The effect sizes (f²) of the individual predictors and their inner-model VIF values are reported in Table 6.

**Table 6:** Hypothesis analysis (Bootstrapping Analysis).

Hypothesis	Original sample (O)	Sample mean (M)	Standard deviation (STDEV)	T statistics (|O/STDEV|)	P values
**Environmental Sustainability Orientation - > BIM-Supported Green Building Adoption**	0.119	0.117	0.037	3.249	0.001
**External Support - > BIM-Supported Green Building Adoption**	0.172	0.168	0.057	3.001	0.003
**Organizational Readiness - > BIM-Supported Green Building Adoption**	0.118	0.118	0.049	2.405	0.016
**Relative Advantage - > BIM-Supported Green Building Adoption**	0.317	0.313	0.049	6.527	0.000
**Technological Competence - > BIM-Supported Green Building Adoption**	0.399	0.393	0.043	9.223	0.000
**Top Management Support - > BIM-Supported Green Building Adoption**	0.378	0.376	0.048	7.955	0.000

Based on Cohen’s effect-size guidelines, Technological Competence produced the strongest effect on BIM-supported green building adoption, followed by Organizational Readiness and Top Management Support. Environmental Sustainability Orientation also demonstrated a medium effect, whereas Relative Advantage and External Support produced small effects. Although Organizational Readiness had a comparatively small standardized path coefficient, its medium f² value indicates that its exclusion would produce a meaningful reduction in the explained variance of BIM-supported green building adoption, suggesting a distinct incremental contribution after accounting for the shared variance among the predictors. All inner-model VIF values ranged from 1.382 to 2.718, remaining below the conservative threshold of 3.3 and indicating that multicollinearity was not a serious concern.

### Bootstrapping based hypothesis analysis

The statistical results of the hypothesis tests presented in Table 7 demonstrate positive and significant correlations for each of the proposed relationships and thus demonstrate a positive relationship to BIM supported green building adoption. Technological competence exhibits the strongest effect (β = 0.399, t = 9.223, p < 0.001), emphasizing that organizations with stronger digital capabilities are more likely to adopt BIM for sustainable construction practices. Top management support also shows a substantial impact (β = 0.378, t = 7.955, p < 0.001), highlighting the critical role of leadership commitment in promoting BIM implementation. Relative advantage demonstrates a strong and significant influence (β = 0.317, t = 6.527, p < 0.001), indicating that perceived benefits of BIM significantly encourage its adoption. External support (β = 0.172, t = 3.001, p = 0.003), environmental sustainability orientation (β = 0.119, t = 3.249, p = 0.001), and organizational readiness (β = 0.118, t = 2.405, p = 0.016) also show positive and significant relationships, although their effects are comparatively smaller. In general, the study’s conclusions show that technology capabilities, managerial commitment, and perceived innovations will be the most important factors influencing BIM-supported green building adoptions. Sustainability orientation and external institutional supports would add additional influence on BIM-supported green building adoptions.

**Table 7:** Effect sizes (f²) and inner-model collinearity (VIF).

Structural Path	f²	Effect	Inner VIF
Technological Competence → Adoption	0.287	Medium	2.436
Top Management Support → Adoption	0.201	Medium	2.718
Relative Advantage → Adoption	0.132	Small	1.964
External Support → Adoption	0.047	Small	1.382
Environmental Sustainability Orientation → Adoption	0.156	Medium	2.105
Organizational Readiness → Adoption	0.231	Medium	2.574

## Discussion

The findings of this study provide important insights into the determinants influencing BIM-supported green building adoption within construction organizations. These findings should be interpreted within the Saudi Arabian institutional and regulatory context in which the data were collected, and generalization to other national settings should be made with caution. Previous research on digital transformation in the construction industry has consistently emphasized that technological capability forms the foundation for successful BIM implementation because it enables organizations to utilize simulation, data integration, and sustainability assessment tools effectively [52,53,63].

This finding is broadly consistent with a recent meta-analysis of 62 BIM adoption studies spanning 13 countries, which found that technical-dimension factors, particularly compatibility with existing workflows, exerted the strongest correlation with BIM adoption among all technical constructs examined (r = 0.555), exceeding that of relative advantage [73,74]. The prominence of technological competence as the leading determinant here reinforces this broader pattern, in which the technical dimension of the TOE framework consistently emerges as a decisive factor across diverse national contexts.

Top management support also demonstrated a strong positive influence on BIM-supported green building adoption. This result reinforces the view that leadership commitment is critical for promoting technological innovation within organizations. The adoption of BIM often requires financial investment, organizational restructuring, and employee training, all of which depend on strategic support from senior management. Prior studies on technology adoption have similarly highlighted that managerial commitment encourages organizational change, facilitates resource allocation, and motivates employees to integrate new digital systems into their workflows [75,76].

A recent large-sample TOE-based study of 512 construction stakeholders in China similarly identified management commitment as one of the two strongest predictors of BIM adoption intention (β = 0.182, p < 0.001), directly corroborating the centrality of leadership support observed in the present study, despite differences in national context and sample composition [77,78].

Relative advantage was found to significantly influence BIM-supported green building adoption, indicating that when organizations perceive BIM as providing operational, collaborative, and sustainability-related benefits, their willingness to adopt the technology increases. This result aligns with innovation diffusion perspectives suggesting that technologies perceived as offering clear performance improvements over traditional practices are more readily adopted [56,62]. BIM enables improved project coordination, enhanced design accuracy, and sustainability analysis, which collectively strengthen its perceived value within construction projects.

This result should, however, be interpreted alongside evidence from the broader BIM adoption literature suggesting that relative advantage, while consistently significant, is not always the dominant technical determinant; a recent meta-analysis found that compatibility with existing workflows exerted a stronger correlation with adoption than relative advantage [78], suggesting that the relative weight of perceived benefits versus workflow fit may vary with the technological maturity of the adopting context.

The findings also reveal that environmental sustainability orientation contributes positively to BIM-supported green building adoption. Organizations that prioritize environmental responsibility in their strategic objectives are more likely to implement digital tools that facilitate sustainable construction practices. BIM technologies provide capabilities for energy analysis, material optimization, and lifecycle performance assessment, which support organizations in achieving their environmental sustainability goals [54,57]. This suggests that sustainability-driven organizational cultures play an important role in accelerating digital transformation within the construction sector.

Direct comparison with prior literature is somewhat limited for this construct, as environmental sustainability orientation is rarely modeled as a discrete determinant in general BIM adoption studies, which have tended to focus on BIM adoption as a technology rather than adoption specifically in service of green building outcomes [78]. This gap underscores the contribution of the present study in isolating sustainability orientation as a distinct antecedent within a green-building-specific adoption model.

External support was also found to have a significant influence on BIM-supported green building adoption, although its effect is relatively smaller compared with internal organizational drivers. Institutional mechanisms such as regulatory encouragement, professional guidance, and industry collaboration help reduce uncertainty associated with technological innovation and provide organizations with the necessary resources and knowledge to implement BIM. Previous research has similarly emphasized the importance of supportive regulatory frameworks and industry networks in promoting the diffusion of digital construction technologies [42,55,61].

This comparatively modest effect mirrors findings from a recent TOE-based study of 512 construction professionals, in which government policy was likewise a significant but comparatively weaker predictor relative to organizational-level factors such as management commitment (β = 0.113 versus β = 0.182, respectively) [73]. Together, these findings suggest that where BIM-specific regulatory mandates remain relatively nascent, internal organizational capabilities may play a proportionally larger role in driving adoption than external institutional pressure.

Finally, organizational readiness demonstrated a positive but comparatively weaker association with BIM-supported green building adoption. This result is noteworthy, because readiness is often theorized as a central enabler of digital transformation. A plausible explanation is that, in the Saudi construction context, readiness may function less as an independent driver and more as a condition that is activated by leadership: where top management support is strong, the resources, training, and structural adjustments that constitute readiness tend to follow, so much of readiness’s influence is channelled through management support rather than exerted directly. The comparatively larger effect size of readiness relative to its path coefficient is consistent with this interpretation, suggesting that readiness contributes meaningfully to explained variance even though its unique direct effect, after accounting for shared variance with other predictors, is modest. This pattern implies that internal preparedness is necessary but, on its own, insufficient to accelerate BIM-supported green building adoption without accompanying leadership commitment and technological competence [79,80].

As with environmental sustainability orientation, organizational readiness is not consistently isolated as a distinct construct in comparable TOE-based BIM adoption studies, which more often subsume readiness-related considerations under broader organizational or training-related factors [73]. This makes direct quantitative comparison difficult and points to an opportunity for future research to examine readiness as a standalone construct across contexts.

Overall, the findings suggest that BIM-supported green building adoption is shaped by a combination of technological capabilities, organizational conditions, and environmental influences. Technological competence, top management support, and perceived relative advantage emerge as the primary drivers, while environmental sustainability orientation, organizational readiness, and external support provide complementary influences. These results reinforce the relevance of integrating technological, organizational, and environmental perspectives when examining digital innovation adoption within the construction industry and demonstrate how BIM can support the transition toward sustainable and smart urban development.

### Limitations and future research directions

Although, the current study provides valuable insights for understanding the factors influencing the use of BIM to support green building development, it has many limitations that should be taken into consideration. The most important limitation is the fact that the empirical evidence was collected through surveys of construction experts from one specific geographic area. Therefore, we cannot generalize the results to other areas of the world (other than those with an analogous climate), nor can we infer the effect of the same variables in various different institutional/industry settings as each area will have their own regulatory framework, level of technological advancement and policies supporting sustainable practices. For example, future researchers could expand upon this study to include multi-country/regional comparative analysis to determine if the factors that are identified here influence the use of BIM in a comparable way across multiple regional/different institutional/industry settings. Moreover, since the empirical evidence was collected using a cross-sectional survey methodology, this type of research design allows for the collection of information regarding perceptions held by organizations at a particular moment. As such, future studies could employ longitudinal research methodologies to further elucidate how the behavior associated with BIM adoption, as well as the capabilities of an organization change throughout the process of transforming itself digitally.

A second major limitation is that this analysis relies entirely upon self-reporting through surveys completed by practitioners within an industry. The results of such analyses are likely to be subject to response bias and/or practitioner’s interpretations of their organization’s actual practice. Therefore, future studies would benefit from using alternative methods for gathering data (e.g., project level performance metrics, case studies, etc.) so as to create a richer context for understanding how BIM supports sustainable construction practices. Additionally, although the study examined important technological, organizational, and environmental influences, there exist other contexts (e.g., organizational culture, regulatory environment, state of digital ecosystems, mechanisms of collaboration among stakeholders) that may affect the use of BIM in sustainable construction projects. Therefore, future research would benefit from expanding the proposed framework with regard to inclusion of these additional context variables, as a means to continue advancing our knowledge base regarding digital innovation adoption, and its role in creating smart and sustainable cities.

### Implications of the study

Theoretically, this study makes three contributions. First, it extends technology-adoption research beyond generic BIM implementation by modeling the determinants of BIM adoption specifically in the green building and sustainability context, showing that environmental sustainability orientation operates as a distinct antecedent alongside conventional TOE factors rather than being subsumed by them. Second, by integrating the TOE framework with DOI’s relative-advantage construct in a single model, it clarifies the previously inconsistent evidence on internal versus external drivers, demonstrating that in the Saudi context technological competence and top management support (internal capabilities) dominate, while external support exerts only a marginal influence. Third, it provides empirical evidence from a rapidly developing, sustainability-focused market that has been underrepresented in prior BIM adoption research, thereby testing the boundary conditions of models developed largely in Western and East Asian settings.

Beyond statistical significance, it is important to consider the practical magnitude of these findings for organizational decision-making, since the effect sizes do not perfectly mirror the ranking of path coefficients. For construction firms, three implications carry the greatest practical weight. First, technological competence produced both the strongest path coefficient and the largest effect size (f² = 0.287), indicating that investment in digital infrastructure, BIM software, and staff technical training is likely to yield the largest practical return in accelerating adoption. Second, despite its comparatively modest path coefficient, organizational readiness carried a medium effect size (f² = 0.231), suggesting that firms should not treat internal readiness-building, such as structured BIM implementation plans and workflow adaptation, as secondary to technology procurement. Third, top management support (f² = 0.201) confirms that leadership commitment remains a practically important lever; firms with limited resources may obtain the greatest practical benefit by prioritizing technological capability and leadership engagement ahead of external-facing initiatives.

External support and environmental sustainability orientation, while statistically significant, showed comparatively smaller practical effects (f² = 0.047 and 0.156, respectively), suggesting that regulatory and institutional interventions alone are unlikely to substitute for firm-level investment in technological and organizational capacity. Because the present study surveyed construction professionals rather than policymakers or urban planners directly, implications for policy and urban planning are offered here as reasoned extensions of the findings rather than as direct empirical conclusions: regulatory encouragement is likely to be most effective when paired with, rather than substituted for, firm-level capacity-building support such as subsidized training or technology-adoption grants.

## Conclusion

This study examined the determinants influencing BIM-supported green building adoption and its contribution to smart and sustainable city development. Consistent with the first objective, the findings demonstrate that technological, organizational, and environmental factors significantly influence the adoption of BIM-supported green building practices within construction organizations. Among these determinants, technological competence, top management support, and relative advantage emerged as the most influential drivers, indicating that strong digital capabilities, leadership commitment, and perceived innovation benefits play critical roles in encouraging BIM adoption. In addition, environmental sustainability orientation, external support, and organizational readiness were also found to contribute positively to BIM implementation, highlighting the importance of sustainability-driven organizational strategies and supportive institutional environments.

Addressing the second objective, the results confirm that managerial support and organizational readiness facilitate the effective implementation of BIM-based sustainable construction strategies. Organizations with supportive leadership and well-prepared internal structures are better positioned to integrate digital technologies into their project management processes and promote environmentally responsible construction practices. Finally, in relation to the third objective, the study situates BIM-supported green building adoption within the broader agenda of smart and sustainable city development. By enabling improved collaboration, lifecycle performance analysis, and sustainability-oriented decision-making, BIM-supported green building practices offer a digital foundation that can support environmentally responsible urban infrastructure development, although this broader link was not tested empirically and represents a direction for future research. Overall, the study highlights the critical role of digital construction technologies in accelerating the transition toward sustainable and smart urban environments.

## Supporting information

S1 Data Dataset used in the study. (XLSX)

S2 File Survey questionnaire. Appendix A contains the demographic questionnaire, and Appendix B contains the main questionnaire and measurement items.
(DOCX)
