# Supplementary material for: BIM-Supported green building adoption in the construction industry
Source: PLoS One. 2026 Sep 15;21(9):e0356894. doi: 10.1371/journal.pone.0356894 (PMC13577526; doi:10.1371/journal.pone.0356894)
Supplement: Supplementary file 1 [file pone.0356894.s002.docx]

Appendix A: Demograpghic Questionnaire

| **Question** | **Option** |
| --- | --- |
| What is your gender? | Male |
|  | Female |
| What is your age group? | 20–29 years |
|  | 30–39 years |
|  | 40–49 years |
|  | 50 years and above |
| What is your highest level of education? | Diploma |
|  | Bachelor’s degree |
|  | Master’s degree |
|  | PhD |
| How many years of professional experience do you have in the construction industry? | Less than 5 years |
|  | 5–10 years |
|  | 11–15 years |
|  | More than 15 years |
| What is your current position in the organization? | Project Engineer |
|  | Site Engineer |
|  | Architect |
|  | BIM Manager / Coordinator |
|  | Project Manager |
|  | Other |
| What type of organization do you work for? | Contractor |
|  | Consultant |
|  | Developer |
|  | Government Agency |
|  | Other |
| What is the size of your organization? | Small (1–50 employees) |
|  | Medium (51–250 employees) |
|  | Large (251+ employees) |
| How long have you been using BIM in your work? | No experience |
|  | Less than 2 years |
|  | 2–5 years |
|  | More than 5 years |
| How many green building projects have you been involved in? | None |
|  | 1–2 projects |
|  | 3–5 projects |
|  | More than 5 projects |

Appendix B: Main Questionnaire

| **Construct and Sources** | **Statement** | **Likert Scale** |
| --- | --- | --- |
| **Technological Competence**  [74], [77], [81], [82], [83] | Our organization has adequate technological infrastructure to support BIM implementation. | 1–5 |
|  | We have access to advanced BIM software and digital tools necessary for construction projects. | 1–5 |
|  | Employees in our organization possess strong technical skills to operate BIM technologies. | 1–5 |
|  | Our organization regularly upgrades its digital technologies to improve BIM capabilities. | 1–5 |
|  | Our IT systems effectively support BIM integration in construction project workflows. | 1–5 |
| **Top Management Support**  [3], [4], [64], [84] | Top management strongly supports the adoption of BIM in our organization. | 1–5 |
|  | Senior management encourages the use of BIM for sustainable construction projects. | 1–5 |
|  | Top management allocates sufficient financial resources for BIM implementation. | 1–5 |
|  | Management promotes employee training related to BIM technologies. | 1–5 |
|  | Organizational leaders emphasize BIM as a strategic priority for sustainability. | 1–5 |
| **Organizational Readiness**  [5], [85], [86], [87], [88] | Our organization is well prepared to adopt BIM in construction projects. | 1–5 |
|  | Employees in our organization are ready to implement BIM-supported green building practices. | 1–5 |
|  | Our organization provides sufficient training programs to support BIM adoption. | 1–5 |
|  | The organizational structure supports BIM integration in project management processes. | 1–5 |
|  | Our organization has clear strategies and plans for BIM implementation. | 1–5 |
| **External Support**  [3], [6], [7], [64], [89] | Government policies encourage the use of BIM in construction projects. | 1–5 |
|  | Industry regulations promote BIM adoption in sustainable building development. | 1–5 |
|  | Professional bodies and industry associations support BIM implementation. | 1–5 |
|  | Government incentives motivate organizations to adopt BIM technologies. | 1–5 |
|  | External stakeholders encourage our organization to use BIM in construction projects. | 1–5 |
| **Environmental Sustainability Orientation**  [2], [90], [91], [92] | Our organization prioritizes environmental sustainability in construction projects. | 1–5 |
|  | We actively promote green building practices within our organization. | 1–5 |
|  | Sustainability goals strongly influence decision-making in our projects. | 1–5 |
|  | Our organization encourages environmentally responsible construction methods. | 1–5 |
|  | Employees in our organization are aware of the environmental benefits of green buildings. | 1–5 |
| **Relative Advantage**  [65], [93], [94], [95], [96] | BIM provides significant advantages compared to traditional construction methods. | 1–5 |
|  | Using BIM improves the efficiency of construction project planning and management. | 1–5 |
|  | BIM enhances collaboration among project stakeholders. | 1–5 |
|  | BIM helps our organization achieve better sustainability outcomes. | 1–5 |
|  | The benefits of BIM adoption outweigh its implementation costs. | 1–5 |
| **BIM-Supported Green Building Adoption**  [97], [98], [99], [100], [101] | Our organization actively uses BIM to support green building development. | 1–5 |
|  | BIM is widely implemented in our organization’s sustainable construction projects. | 1–5 |
|  | Our organization plans to increase BIM usage in future green building projects. | 1–5 |
|  | BIM plays a key role in achieving sustainable building design in our projects. | 1–5 |
|  | Our organization encourages the use of BIM for environmentally sustainable construction. | 1–5 |
