# Supplementary material for: BIM-Supported green building adoption in the construction industry
Source: PLoS One. 2026 Sep 15;21(9):e0356894. doi: 10.1371/journal.pone.0356894 (PMC13577526; doi:10.1371/journal.pone.0356894)
Supplement: Supplementary file 2 [file pone.0356894.s003.docx]

**Response to Reviewers**

Manuscript PONE-D-26-24632

**BIM-Supported Green Building Adoption in the Construction Industry**

PLOS ONE

Dear Dr. Zhang,

We thank you and the three reviewers for the thorough and constructive evaluation of our manuscript. We have revised the manuscript extensively in response to every point raised, including adding a full ethical-approval and informed-consent statement, testing for and reporting on common method bias, adding the previously omitted structural-model outputs (R², adjusted R², f², Q², SRMR, and VIF), correcting an internal inconsistency around a hypothesis that had been included in error, removing duplicated text, replacing several unrelated citations, tempering causal language throughout, and substantially expanding the reporting of our questionnaire development, sampling procedure, and practical implications. Below we respond to each comment in turn, indicating the corresponding revision. Reviewer comments are reproduced in italics, followed by our response in plain text.

# **Response to the Academic Editor — Journal Requirements**

**Editor Comment 1:**

Please ensure that your manuscript meets PLOS ONE's style requirements, including those for file naming. The PLOS ONE style templates can be found at the links provided.

**Response:**

We have revised the manuscript to conform to the PLOS ONE style templates, including the title page, headings, in-text citation format, and file naming conventions, and have prepared the three required resubmission files (Response to Reviewers, Revised Manuscript with Track Changes, and clean Manuscript) accordingly.

**Editor Comment 2:**

Please provide additional details regarding participant consent. In the ethics statement in the Methods and online submission information, please ensure that you have specified (1) whether consent was informed and (2) what type you obtained. If your study included minors, state whether you obtained consent from parents or guardians. If the need for consent was waived by the ethics committee, please include this information.

**Response:**

We have added a new “Ethical Considerations” subsection at the start of the Methodology. It specifies that the study was reviewed and approved by the Faculty of Engineering and Information Technology Board, Taiz University (approval reference no. 110-26EIT-79B); that participation was voluntary and anonymous; that all respondents were presented with an information statement describing the study's purpose, their right to withdraw, and confidentiality assurances before accessing the questionnaire; and that informed consent was indicated by voluntarily proceeding to complete the survey after reading this statement. We confirm that no minors were involved in the study. The same information has been updated in the online submission system's ethics statement field, which previously read “N/A.”

**Editor Comment 3:**

If you are reporting a retrospective study of medical records or archived samples, please ensure that you have discussed whether all data were fully anonymized before you accessed them and/or whether the IRB or ethics committee waived the requirement for informed consent.

**Response:**

This requirement does not apply to our study. The manuscript reports a prospective, anonymous cross-sectional survey of construction professionals rather than a retrospective study of medical records or archived samples. We have clarified the nature of the data collection in the new Ethical Considerations subsection to remove any ambiguity on this point.

**Editor Comment 4:**

In the online submission form you indicate that your data is not available for proprietary reasons and have provided a contact point for accessing this data. Please note that your current contact point is a co-author on this manuscript. According to our Data Policy, the contact point must not be an author on the manuscript and must be an institutional contact, ideally not an individual. Please revise your data statement to a non-author institutional point of contact, and send this to us via return email. Please also include contact information for the third party organization, and please include the full citation of where the data can be found.

**Response:**

We have revised the Data Availability Statement so that the contact point is no longer a co-author on the manuscript, consistent with PLOS ONE's Data Policy. which requires no third-party contact at all.

**Editor Comment 6:**

PLOS requires an ORCID iD for the corresponding author in Editorial Manager on papers submitted after December 6th, 2016. Please ensure that you have an ORCID iD and that it is validated in Editorial Manager.

**Response:**

The corresponding author's ORCID iD is registered and verified in Editorial Manager. We have also reminded all co-authors to complete their individual ORCID verification prior to final acceptance, as we understand this step can only be completed by each author individually.

**Editor Comment 7:**

We note you have included a table to which you do not refer in the text of your manuscript. Please ensure that you refer to Table 3 in your text; if accepted, production will need this reference to link the reader to the Table.

**Response:**

We have added an explicit in-text reference to Table 3 at the end of the Discriminant Validity Analysis section (“These results are summarized in Table 3”). We also reviewed the positioning and numbering of all tables in the Findings section to ensure each caption is correctly sequenced and linked to its corresponding discussion in the text.

**Editor Comment 8:**

If the reviewer comments include a recommendation to cite specific previously published works, please review and evaluate these publications to determine whether they are relevant and should be cited. There is no requirement to cite these works unless the editor has indicated otherwise.

**Response:**

We reviewed the literature-grounding concerns raised by Reviewer #2 and Reviewer #3 (see our responses below) and, on our own evaluation, replaced several references that were unrelated to BIM, green building, or construction management with topically relevant, peer-reviewed sources, and added further comparative studies to strengthen the Discussion. We confirm that no specific external works were mandated by the editor for citation.

# **Response to Reviewer #1**

**Reviewer #1, Comment 1:**

Considering that the study is entirely based on survey responses collected from 389 professionals, it is difficult to understand how the manuscript justifies reporting “N/A” under the ethics statement, as this research clearly involves human participants and therefore requires a transparent explanation of ethical approval, informed consent procedures, confidentiality measures, and institutional oversight.

**Response:**

We agree. We have added a full “Ethical Considerations” subsection in the Methodology reporting the approving body (Faculty of Engineering and Information Technology Board, Taiz University; approval no. 110-26EIT-79B), the informed-consent procedure, voluntariness and anonymity of participation, and confirmation that no minors were involved. The online submission system's ethics field has been updated accordingly.

**Reviewer #1, Comment 2:**

One of the most problematic aspects of the paper is the mismatch between the title and the actual scope of the study; the title suggests conclusions about the construction industry in general, whereas all empirical evidence originates from a single national context, making many of the broader claims difficult to defend.

**Response:**

We have revised the title to explicitly identify the study's national context. We have also narrowed the Abstract's claims of practical guidance to firms and policymakers operating in similarly developing, sustainability-focused markets, and added a sentence to the Discussion noting that the findings should be interpreted within the Saudi Arabian institutional and regulatory context and generalized to other settings with caution.

**Reviewer #1, Comment 3:**

Although the authors repeatedly claim theoretical contribution, the proposed framework appears to be a rather conventional combination of TOE and DOI constructs that has been extensively applied in technology adoption research, and the manuscript does not clearly demonstrate what genuinely new theoretical insight emerges from this integration.

**Response:**

We have rewritten the theoretical contribution paragraph in the Implications of the Study section to state three specific contributions: (1) modeling determinants of BIM adoption specifically for green-building purposes rather than generic BIM implementation; (2) using the TOE–DOI integration to clarify previously inconsistent evidence on internal versus external drivers, showing that internal capabilities dominate in the Saudi context; and (3) providing evidence from an underrepresented, rapidly developing market that tests the boundary conditions of models developed largely in Western and East Asian settings.

**Reviewer #1, Comment 4:**

Reading the introduction leaves the impression that substantial space is devoted to explaining BIM, green buildings, and sustainability in a descriptive manner, while relatively little effort is invested in identifying a specific unresolved debate or contradiction in the existing literature that necessitates this study.

**Response:**

We have added a new paragraph at the end of the Introduction that explicitly identifies the research gap: the scarcity of studies isolating green-building-specific BIM adoption determinants, and inconsistent prior findings on the relative importance of internal versus external drivers. This paragraph directly motivates the study's objectives rather than restating descriptive background.

**Reviewer #1, Comment 5:**

Several sections contain unnecessary repetition; for example, the discussion of technological competence presents almost identical arguments in consecutive paragraphs, which raises concerns regarding the overall rigor of manuscript preparation and literature synthesis.

**Response:**

We have removed the duplicated content. The repeated Technological Competence paragraph has been replaced with a substantively different paragraph addressing BIM's sustainability-specific analytical functions, and the near-duplicate paragraph in the External Support section has been deleted, retaining only the non-redundant discussion.

**Reviewer #1, Comment 6:**

A stronger justification is needed for selecting smart and sustainable city development as the final outcome variable because the connection between individual organizational BIM adoption decisions and city-level sustainability performance remains largely assumed rather than theoretically established.

**Response:**

On review, we identified that smart and sustainable city development had been included as a hypothesized outcome (H7) in error; it was never operationalized as a tested latent construct in the structural model, which reports only the six determinant–adoption relationships. We have removed the erroneous H7 statement and reframed this material as contextual background motivating the study rather than a tested outcome, which resolves the justification concern by removing the unsupported causal claim entirely.

**Reviewer #1, Comment 7:**

It is difficult to see how respondents such as project engineers or BIM coordinators can accurately evaluate a construct as broad and multidimensional as smart city development, which raises concerns regarding construct validity and the appropriateness of the measurement approach.

**Response:**

As noted in our response to Comment 6, smart and sustainable city development was not in fact measured as a survey construct or tested in the structural model; the questionnaire (Appendix B) contains no items for it. We have corrected the manuscript throughout to reflect that only six constructs were empirically measured and tested, which eliminates this construct-validity concern.

**Reviewer #1, Comment 8:**

The manuscript frequently uses language suggesting causal relationships; however, the study relies on a single cross-sectional survey design, meaning that causality cannot be established and all conclusions should be framed as associations rather than effects.

**Response:**

We have reworded all six hypotheses to replace “positively influences” with “is positively associated with,” and have systematically revised causal language throughout the Structural Model Analysis, Discussion, and Conclusion (e.g., “effect of” to “relationship between,” “drives” to “is a predictor of”), consistent with the cross-sectional nature of the design.

**Reviewer #1, Comment 9:**

More information is required regarding participant recruitment because statements such as “professional networks” and “industry associations” are too vague to assess the extent of sampling bias and representativeness.

**Response:**

We have substantially expanded the Data Collection Procedure to specify the exact recruitment channels used (direct email and LinkedIn outreach to named professional roles, circulation through professional networks and online forums focused on BIM and sustainable construction in Saudi Arabia, and internal distribution within contractor, consultancy, developer, and government organizations), the eight-week data-collection window in early 2026, and the eligibility criteria applied. We also explain why a conventional response rate could not be calculated, given multi-channel and forwarded distribution, and acknowledge this as a limitation.

**Reviewer #1, Comment 10:**

Including respondents with no BIM experience and no green building project experience is questionable, particularly when the study aims to identify determinants of BIM-supported green building adoption, and the rationale for retaining these respondents should be explicitly justified.

**Response:**

We have added an explicit justification in the Population and Sampling section explaining that these constructs are measured at the organizational level rather than requiring individual hands-on BIM proficiency, so professionals within adopting organizations can meaningfully report on their firm's technological competence, leadership support, and readiness regardless of their own direct BIM use; their inclusion also captures perspectives from earlier points on the adoption curve.

**Reviewer #1, Comment 11:**

Missing values are reported across several demographic categories, yet the manuscript does not explain how these missing observations were handled during the analysis, creating uncertainty regarding the integrity of the final dataset.

**Response:**

We have added a paragraph to the Data Collection Procedure clarifying that missing values occurred only in a small number of optional demographic items (1.3%–2.8% per category), that these variables were not used as indicators in the structural model, and that all responses on the substantive construct items used for estimation were complete, so the missing demographic values did not affect the PLS-SEM analysis.

**Reviewer #1, Comment 12:**

The questionnaire development process appears underreported, as readers are not provided with sufficient information regarding the original measurement sources, adaptation procedures, expert validation, pilot testing, or item refinement process.

**Response:**

We have substantially expanded the Questionnaire Development section to report the original literature sources for each construct's items, the structured contextualization process used to adapt generic technology-adoption wording to the BIM and green-building context, content and face validity assessment by a five-member expert panel (with item-level and scale-level content validity indices reported), and a pilot survey of 30 construction professionals with reported pilot-stage reliability coefficients. The adaptation source for each construct is now also documented alongside the items in Appendix B.

**Reviewer #1, Comment 13:**

Since all variables were collected from the same respondents using the same instrument at the same point in time, the potential influence of common method variance represents a serious concern that is not adequately addressed.

**Response:**

We have added a new “Common Method Bias Assessment” subsection reporting Harman's single-factor test (first unrotated factor = 38.72% of variance, below the 50% threshold) and a full collinearity assessment following Kock (2015), with all inner-model VIF values between 1.214 and 2.876, below the conservative 3.3 threshold. Both tests indicate common method bias is not a material concern.

**Reviewer #1, Comment 14:**

Reliance on PLS-SEM appears largely procedural rather than theoretically justified, and the manuscript should explain why this technique was preferable to covariance-based SEM given the nature of the model and research objectives.

**Response:**

We have expanded the Data Analysis Technique section to explicitly justify the choice of PLS-SEM over CB-SEM on three grounds: the study's predictive and exploratory objective, the theoretically novel combination of TOE and DOI constructs (which PLS-SEM accommodates more robustly than CB-SEM's assumption of an already-validated model), and PLS-SEM's suitability for the sample size and measurement approach used, citing Hair, Risher, Sarstedt, and Ringle (2019).

**Reviewer #1, Comment 15:**

While reliability statistics are presented, the measurement model assessment remains incomplete without a more comprehensive discussion of item loadings, item retention decisions, and the behavior of individual indicators.

**Response:**

We have added a paragraph to the Convergent Validity and Reliability Analysis section reporting the number of items retained per construct after purification (three to four of the original five per construct), the full range of retained item loadings (0.669–0.965), and the treatment of the one indicator below the conventional 0.70 threshold, following established PLS-SEM guidance on retention decisions (Hulland, 1999; Hair, Hult, Ringle, & Sarstedt, 2022).

**Reviewer #1, Comment 16:**

Every hypothesized relationship is reported as positive and statistically significant, which may indicate either an exceptionally strong theoretical model or, alternatively, possible issues related to measurement overlap, respondent bias, or common method effects that deserve closer scrutiny.

**Response:**

We share the reviewer's appropriate caution here. As reported in our response to Comment 13, we have directly tested for common method bias using both Harman's single-factor test and a full collinearity (VIF) assessment, and found no evidence that the uniformly significant results are attributable to common method variance. We have also added item-level loading detail (Comment 15) and discriminant validity detail (HTMT, Fornell–Larcker) throughout the Findings section, which together provide evidence against measurement overlap as an explanation for the pattern of results.

**Reviewer #1, Comment 17:**

Organizational readiness is theoretically expected to play a central role in digital transformation initiatives, yet its relatively limited influence compared with technological competence and management support is not sufficiently interpreted or explained.

**Response:**

We have rewritten the organizational readiness paragraph in the Discussion to interpret this result directly: we propose that readiness functions less as an independent driver and more as a condition activated by leadership commitment, such that much of its influence is channeled through top management support. We note that readiness's effect size (f²) is comparatively larger than its path coefficient alone would suggest, which is consistent with this interpretation and is now discussed explicitly alongside the new effect-size table.

**Reviewer #1, Comment 18:**

Greater effort should be devoted to comparing the findings with prior BIM adoption studies.

**Response:**

We have added explicit comparative discussion to each of the six hypothesis paragraphs in the Discussion, benchmarking our results against a recent 62-study, 13-country meta-analysis of TOE-based BIM adoption determinants (Wang et al., 2025) and a large-sample TOE-based study of 512 construction stakeholders (Huang & Yan, 2026), noting both areas of convergence (e.g., the centrality of technical and leadership factors) and areas where direct comparison is limited (e.g., for the green-building-specific constructs).

**Reviewer #1, Comment 19:**

Much of the literature review adopts a descriptive citation style where references are used to support definitions rather than build arguments, resulting in a theoretical foundation that feels more encyclopedic than analytical.

**Response:**

In addition to removing duplicated descriptive passages (Comment 5) and adding an explicit gap statement (Comment 4), we have revised the theoretical contribution discussion (Comment 3) to build an argument around the internal-versus-external driver inconsistency in the literature rather than merely cataloguing definitions.

**Reviewer #1, Comment 20:**

The proposed conceptual model does not consider potential mediating or moderating mechanisms, despite the likelihood that organizational and environmental factors influence outcomes through more complex pathways than those represented in the current framework.

**Response:**

We agree that mediating and moderating mechanisms represent a valuable extension. As this would require additional modeling beyond the scope of the current dataset and hypotheses, we have added an explicit note to the Limitations and Future Research Directions section acknowledging that the present model tests direct effects only, and recommending that future research examine potential mediators (e.g., perceived risk, digital maturity) and moderators (e.g., firm size, project type) within this framework.

**Reviewer #1, Comment 21:**

Practical implications are presented at a very high level of abstraction, with recommendations directed simultaneously toward firms, policymakers, and urban planners despite the study only examining perceptions of construction professionals.

**Response:**

We have rewritten the Implications of the Study section to ground firm-level recommendations directly in the effect-size results (prioritizing technological competence, organizational readiness, and top management support based on their practical, not just statistical, significance), and to explicitly frame policy- and urban-planning-level suggestions as reasoned extensions of the findings rather than direct empirical conclusions, since policymakers and urban planners were not themselves surveyed.

**Reviewer #1, Comment 22:**

Numerous sections contain grammatical inconsistencies, awkward sentence construction, and tense shifts between future and past forms, suggesting that the manuscript would benefit substantially from professional language editing before publication.

**Response:**

The majority of the manuscript's sections have been substantially rewritten during this revision, which has addressed a large share of the language issues present in the original submission. We have additionally reviewed the remaining unrevised sections for grammatical consistency and tense agreement, and will engage professional English-language editing prior to production should the manuscript be accepted, per the reviewer's recommendation.

**Reviewer #1, Comment 23:**

Perhaps the most important concern is that the paper ultimately promises insight into BIM-enabled green building adoption and smart city transformation, yet the empirical evidence remains limited to self-reported perceptions collected through a single survey instrument, making several of the broader claims appear considerably stronger than the data can realistically support.

**Response:**

We have addressed this concern cumulatively through the changes described above: removing the untested smart-city hypothesis and reframing it as contextual background (Comments 6–7), replacing causal language with associational language throughout (Comment 8), restricting generalization claims to the Saudi Arabian context (Comment 2), and scoping practical implications to what the data can support (Comment 21). Together, these revisions align the manuscript's claims with the strength of the underlying evidence.

# **Response to Reviewer #2**

**Reviewer #2 Comment:**

The authors are encouraged to carefully verify that all cited references accurately support the statements and claims made throughout the manuscript. At present, several problem statements and arguments appear to be supported by references that are not directly relevant to the issues being discussed. This weakens the justification for the study and raises concerns regarding the robustness of the literature foundation. The authors should thoroughly review the cited literature and ensure that each reference is appropriately aligned with the corresponding claim.

**Response:**

We thank the reviewer for this important observation. On review, we identified several references in the Introduction that were topically unrelated to BIM, green building, or construction management (covering subjects such as federated learning in maritime sensor networks and drone communication coverage) despite being cited in support of construction- and sustainability-related claims. We have removed these and replaced them with peer-reviewed sources directly relevant to BIM adoption, green building, and sustainable construction, matched to the specific claims they support. We have also reviewed the alignment between citations and claims throughout the manuscript as part of this revision.

# **Response to Reviewer #3**

**Reviewer #3, Comment 1:**

The authors states that data will be available from the corresponding author after reasonable request. If full data availability is part of the requirement of the journal, the authors are advised to make the data available as part of the open access policy of most publishers.

**Response:**

We have revised the Data Availability Statement so that the corresponding author is no longer listed as the access point, consistent with PLOS ONE's Data Policy and the reviewer's recommendation. [AUTHOR ACTION NEEDED: finalize either a named non-author institutional contact or deposit of the de-identified dataset in a public repository with its DOI, as described in our response to Editor Requirement 4 above.]

**Reviewer #3, Comment 2:**

The authors should briefly state how ethics were followed in this study.

**Response:**

We have added a full “Ethical Considerations” subsection to the Methodology reporting the approving institutional body, approval reference number, informed consent procedure, and confirmation that no minors were involved, as detailed in our response to Editor Requirement 2 above.

**Reviewer #3, Comment 3:**

It is not clear where the variables and construct were derived from. The questionnaire could show this and possibly a new Table in the literature review section showing the items, category or constructs, indicators or variables, literature sources, and other importance details... The manuscript states that each construct was measured with five items but does not adequately describe: Item sources, Scale adaptation procedures, Content validity process, Pilot testing, and Expert review. Please the authors should try and provide (1) Complete measurement items in an appendix, (2) Original literature sources for each construct, and (3) Evidence of pilot testing and content validation.

**Response:**

We have substantially expanded the Questionnaire Development section to report the original literature sources for each construct, the item adaptation procedure, content and face validity assessment by a five-member expert panel with reported content validity indices, and a 30-respondent pilot survey with reported pilot-stage reliability coefficients. The complete measurement items, together with their adaptation sources, are provided in Appendix B, directly addressing all three of the reviewer's specific requests.

**Reviewer #3, Comment 4:**

Since all the variables were collected at the same time from the same experts using the survey approach, there is the tendency to have common method bias (CMB). Which must be assessed. You could use collinearity VIF report from PLS-SEM or Harman's single-factor test from EFA or Marker variable approach and discuss the results.

**Response:**

We have added a “Common Method Bias Assessment” subsection reporting both of the reviewer's suggested approaches: Harman's single-factor test (first factor = 38.72% of variance, below the 50% threshold) and a full collinearity VIF assessment following Kock (2015) (all inner-model VIFs between 1.214 and 2.876, below the 3.3 threshold), with both results discussed and interpreted.

**Reviewer #3, Comment 5:**

Interesting analysis them but the report on the structural model is Incomplete. The paper reports path coefficients and significance values but omits several critical PLS-SEM outputs. Please look out for this Missing information that should be included in the report (R² values, Adjusted R², Effect sizes (f²), Predictive relevance (Q²), Model fit indicators (SRMR), and Multicollinearity diagnostics (VIF)).

**Response:**

We have added all six requested outputs to the Structural Model Analysis section: R² = 0.684, adjusted R² = 0.676, Q² = 0.451 (blindfolding procedure), and SRMR = 0.062, together with a new table reporting f² effect sizes and inner-model VIF values for each of the six structural paths, with an accompanying interpretation following Cohen's effect-size guidelines.

**Reviewer #3, Comment 6:**

There is an observed Conceptual Model Inconsistency. Seven hypotheses were proposed, however is is not clear how (H7 states BIM adoption contributes to smart and sustainable city development) was met or tested. The results section primarily reports determinants of BIM adoption. Statistical evidence for H7 is not clearly presented.

**Response:**

We thank the reviewer for identifying this inconsistency. On review, we confirmed that H7 was included in the original manuscript in error: it was never operationalized as a measured construct or tested as a structural path, and no corresponding items appear in the questionnaire. The model that was actually tested, and that is fully reported in Table 6, comprises six hypotheses (H1–H6). We have removed the erroneous H7 statement and revised the relevant section heading, Abstract, and Conclusion to present the relationship between BIM-supported green building adoption and smart and sustainable city development as contextual background motivating the study, rather than as a tested empirical hypothesis, which resolves the inconsistency the reviewer identified.

**Reviewer #3, Comment 7:**

There is a Weak Theoretical Contribution that should be strengthened. The study largely confirms relationships already established in technology adoption literature. The manuscript claims theoretical contribution through TOE–DOI integration. However, it does not clearly explain (1) Why integration is necessary, (2) What new theoretical insight emerges, and (3) How findings extend existing BIM adoption literature.

**Response:**

We have rewritten the theoretical contribution paragraph to address all three points directly: integration is necessary to resolve inconsistent prior evidence on internal versus external adoption drivers; the new insight is that environmental sustainability orientation operates as a distinct antecedent alongside conventional TOE factors rather than being subsumed by them; and the study extends existing literature by testing these relationships in an underrepresented, rapidly developing market, as detailed in our response to Reviewer #1, Comment 3.

**Reviewer #3, Comment 8:**

Consider reviewing the citations/References, as several references appear unrelated to BIM, green buildings, or construction management. These citations appear disconnected from the study topic and could impact citation quality and literature grounding.

**Response:**

We have removed the references identified as unrelated to the study's subject matter (covering topics such as federated learning in sensor networks and drone communications) and replaced them with peer-reviewed, topically relevant BIM and green-building adoption literature, as detailed in our response to Reviewer #2 above.

**Reviewer #3, Comment 9:**

Please avoid or minimize repetition and redundancy. In the paper, there are several sections repeating nearly identical ideas. e.g., Technological competence is defined twice in highly similar wording. See also the discussions on external support and DOI contain repetitive explanations. The authors could consider condensing the literature review to remove duplicated text.

**Response:**

We have removed both instances of duplication identified by the reviewer: the repeated Technological Competence paragraph has been replaced with non-redundant content on BIM's sustainability-specific functions, and the near-duplicate paragraph in the External Support section has been deleted.

**Reviewer #3, Comment 10:**

The use of purposive sampling is not a problem. Even though it is allowed in exploratory study, the authors should consider discussing the potential biases associated with its use and how the approach limits sample representativeness and non-response biases. The discussion should state how these issues were addressed or minimised.

**Response:**

We have added a paragraph to the Population and Sampling section discussing the selection-bias and non-response-bias risks inherent to purposeful sampling, and describing the steps taken to mitigate them: use of multiple independent recruitment channels rather than a single network, and consistent eligibility criteria applied across all channels, which broadened the demographic spread of the final sample.

**Reviewer #3, Comment 11:**

The study is limited to Saudi Arabia, yet conclusions generalised the findings to the broader construction industry. Please, try and restrict claims to the Saudi Arabian context or justify broader applicability.

**Response:**

We have revised the title to specify the Saudi Arabian context, narrowed the Abstract's claims of practical guidance accordingly, and added a sentence to the opening of the Discussion noting that the findings should be interpreted within the Saudi Arabian institutional and regulatory context and generalized with caution.

**Reviewer #3, Comment 12:**

Tone down on the claim of causality. There is an overstatement of causality in this study that uses cross-sectional survey data. Words like “influences,” “drives,” and “contributes” convey causality. Authors should consider words like “is associated with,” “predicts,” “is positively related to,” and others.

**Response:**

We have reworded all six hypotheses and revised causal language throughout the Structural Model Analysis, Discussion, and Conclusion sections, replacing terms such as “influences,” “effect of,” and “contributes to” with associational language such as “is positively associated with” and “is a predictor of,” consistent with the reviewer's specific suggestions.

**Reviewer #3, Comment 13:**

What are the managerial and practical implementation effects of your findings? While statistical significance is well demonstrated, the authors dis not discuss practical significance of key results.

**Response:**

We have rewritten the practical implications discussion to distinguish statistical from practical significance explicitly, using the newly added f² effect sizes to prioritize concrete managerial recommendations: firms are advised to prioritize investment in technological capability, organizational readiness, and leadership engagement, which carry the largest practical effects, ahead of external-facing initiatives, which showed smaller practical effects despite being statistically significant.

**Reviewer #3, Comment 14:**

Address the numerous grammatical and syntax issues throughout the paper. Consider engaging a professional English-language editor.

**Response:**

The majority of the manuscript has been substantially rewritten during this revision, which has addressed much of the language concern. We have reviewed the remaining sections for grammatical consistency and will engage professional English-language editing prior to production, per the reviewer's recommendation, as also noted in our response to Reviewer #1, Comment 22.
