# Supplementary material for: BIM-Supported green building adoption in the construction industry
Source: PLoS One. 2026 Sep 15;21(9):e0356894. doi: 10.1371/journal.pone.0356894 (PMC13577526; doi:10.1371/journal.pone.0356894)
Supplement: Peer review history

# Peer review: Decision Letter 0

**DOI:** 10.1371/journal.pone.0356894.r001

**Authors:** Peng Zhang

16 Jun 2026

Dear Dr. Alawag,

Thank you for submitting your manuscript to PLOS ONE. After careful consideration, we feel that it has merit but does not fully meet PLOS ONE’s publication criteria as it currently stands. Therefore, we invite you to submit a revised version of the manuscript that addresses the points raised during the review process.

Please submit your revised manuscript by Aug 15 2026 11:59PM. If you will need more time than this to complete your revisions, please reply to this message or contact the journal office at plosone@plos.org. When you're ready to submit your revision, log on to https://www.editorialmanager.com/pone/ and select the 'Submissions Needing Revision' folder to locate your manuscript file.

- A letter that responds to each point raised by the academic editor and reviewer(s). You should upload this letter as a separate file labeled 'Response to Reviewers'.
- A marked-up copy of your manuscript that highlights changes made to the original version. You should upload this as a separate file labeled 'Revised Manuscript with Track Changes'.
- An unmarked version of your revised paper without tracked changes. You should upload this as a separate file labeled 'Manuscript'.

If you would like to make changes to your financial disclosure, please include your updated statement in your cover letter. Guidelines for resubmitting your figure files are available below the reviewer comments at the end of this letter.

If applicable, we recommend that you deposit your laboratory protocols in protocols.io to enhance the reproducibility of your results. Protocols.io assigns your protocol its own identifier (DOI) so that it can be cited independently in the future. For instructions see: https://journals.plos.org/plosone/s/submission-guidelines#loc-laboratory-protocols. Additionally, PLOS ONE offers an option for publishing peer-reviewed Lab Protocol articles, which describe protocols hosted on protocols.io. Read more information on sharing protocols at https://plos.org/protocols?utm_medium=editorial-email&utm_source=authorletters&utm_campaign=protocols.

As the corresponding author, your ORCID iD is verified in the submission system and will appear in the published article. PLOS supports the use of ORCID, and we encourage all coauthors to register for an ORCID iD and use it as well. Please encourage your coauthors to verify their ORCID iD within the submission system before final acceptance, as unverified ORCID iDs will not appear in the published article. *Only*  the individual author can complete the verification step; PLOS staff *cannot*  verify ORCID iDs on behalf of authors.

We look forward to receiving your revised manuscript.

Kind regards,

Peng Zhang, Ph.D.

Academic Editor

PLOS One

**Journal Requirements:**

When submitting your revision, we need you to address these additional requirements.

1. Please ensure that your manuscript meets PLOS ONE’s style requirements, including those for file naming. The PLOS ONE style templates can be found at

https://journals.plos.org/plosone/s/file?id=wjVg/PLOSOne_formatting_sample_main_body.pdf and

https://journals.plos.org/plosone/s/file?id=ba62/PLOSOne_formatting_sample_title_authors_affiliations.pdf

2. Please provide additional details regarding participant consent. In the ethics statement in the Methods and online submission information, please ensure that you have specified (1) whether consent was informed and (2) what type you obtained (for instance, written or verbal, and if verbal, how it was documented and witnessed). If your study included minors, state whether you obtained consent from parents or guardians. If the need for consent was waived by the ethics committee, please include this information.

3. If you are reporting a retrospective study of medical records or archived samples, please ensure that you have discussed whether all data were fully anonymized before you accessed them and/or whether the IRB or ethics committee waived the requirement for informed consent. If patients provided informed written consent to have data from their medical records used in research, please include this information.

4. In the online submission form you indicate that your data is not available for proprietary reasons and have provided a contact point for accessing this data. Please note that your current contact point is a co-author on this manuscript. According to our Data Policy, the contact point must not be an author on the manuscript and must be an institutional contact, ideally not an individual. Please revise your data statement to a non-author institutional point of contact, such as a data access or ethics committee, and send this to us via return email. Please also include contact information for the third party organization, and please include the full citation of where the data can be found.

6. PLOS requires an ORCID iD for the corresponding author in Editorial Manager on papers submitted after December 6th, 2016. Please ensure that you have an ORCID iD and that it is validated in Editorial Manager. To do this, go to ‘Update my Information’ (in the upper left-hand corner of the main menu), and click on the Fetch/Validate link next to the ORCID field. This will take you to the ORCID site and allow you to create a new iD or authenticate a pre-existing iD in Editorial Manager.

7. We note you have included a table to which you do not refer in the text of your manuscript. Please ensure that you refer to Table 3 in your text; if accepted, production will need this reference to link the reader to the Table.

8. If the reviewer comments include a recommendation to cite specific previously published works, please review and evaluate these publications to determine whether they are relevant and should be cited. There is no requirement to cite these works unless the editor has indicated otherwise.

[Note: HTML markup is below. Please do not edit.]

Reviewers' comments:

Reviewer’s Responses to Questions

**Comments to the Author**

1. Is the manuscript technically sound, and do the data support the conclusions?

Reviewer #1: Partly

Reviewer #2: No

Reviewer #3: Yes

**********

2. Has the statistical analysis been performed appropriately and rigorously?

Reviewer #1: Yes

Reviewer #2: No

Reviewer #3: Yes

**********

3. Have the authors made all data underlying the findings in their manuscript fully available?

The PLOS Data policy

Reviewer #1: Yes

Reviewer #2: Yes

Reviewer #3: No

**********

4. Is the manuscript presented in an intelligible fashion and written in standard English?

Reviewer #1: Yes

Reviewer #2: Yes

Reviewer #3: Yes

**********

Reviewer #1: This manuscript investigates the determinants of BIM-supported green building adoption and its contribution to smart and sustainable city development; however, I have several serious concerns that must be satisfactorily addressed before the manuscript can be considered for publication.

1. Considering that the study is entirely based on survey responses collected from 389 professionals, it is difficult to understand how the manuscript justifies reporting "N/A" under the ethics statement, as this research clearly involves human participants and therefore requires a transparent explanation of ethical approval, informed consent procedures, confidentiality measures, and institutional oversight.

2. One of the most problematic aspects of the paper is the mismatch between the title and the actual scope of the study; the title suggests conclusions about the construction industry in general, whereas all empirical evidence originates from a single national context, making many of the broader claims difficult to defend.

3. Although the authors repeatedly claim theoretical contribution, the proposed framework appears to be a rather conventional combination of TOE and DOI constructs that has been extensively applied in technology adoption research, and the manuscript does not clearly demonstrate what genuinely new theoretical insight emerges from this integration.

4. Reading the introduction leaves the impression that substantial space is devoted to explaining BIM, green buildings, and sustainability in a descriptive manner, while relatively little effort is invested in identifying a specific unresolved debate or contradiction in the existing literature that necessitates this study.

5. Several sections contain unnecessary repetition; for example, the discussion of technological competence presents almost identical arguments in consecutive paragraphs, which raises concerns regarding the overall rigor of manuscript preparation and literature synthesis.

6. A stronger justification is needed for selecting smart and sustainable city development as the final outcome variable because the connection between individual organizational BIM adoption decisions and city-level sustainability performance remains largely assumed rather than theoretically established.

7. It is difficult to see how respondents such as project engineers or BIM coordinators can accurately evaluate a construct as broad and multidimensional as smart city development, which raises concerns regarding construct validity and the appropriateness of the measurement approach.

8. The manuscript frequently uses language suggesting causal relationships; however, the study relies on a single cross-sectional survey design, meaning that causality cannot be established and all conclusions should be framed as associations rather than effects.

9. More information is required regarding participant recruitment because statements such as "professional networks" and "industry associations" are too vague to assess the extent of sampling bias and representativeness.

10. Including respondents with no BIM experience and no green building project experience is questionable, particularly when the study aims to identify determinants of BIM-supported green building adoption, and the rationale for retaining these respondents should be explicitly justified.

11. Missing values are reported across several demographic categories, yet the manuscript does not explain how these missing observations were handled during the analysis, creating uncertainty regarding the integrity of the final dataset.

12. The questionnaire development process appears underreported, as readers are not provided with sufficient information regarding the original measurement sources, adaptation procedures, expert validation, pilot testing, or item refinement process.

13. Since all variables were collected from the same respondents using the same instrument at the same point in time, the potential influence of common method variance represents a serious concern that is not adequately addressed.

14. Reliance on PLS-SEM appears largely procedural rather than theoretically justified, and the manuscript should explain why this technique was preferable to covariance-based SEM given the nature of the model and research objectives.

15. While reliability statistics are presented, the measurement model assessment remains incomplete without a more comprehensive discussion of item loadings, item retention decisions, and the behavior of individual indicators.

16. Every hypothesized relationship is reported as positive and statistically significant, which may indicate either an exceptionally strong theoretical model or, alternatively, possible issues related to measurement overlap, respondent bias, or common method effects that deserve closer scrutiny.

17. Organizational readiness is theoretically expected to play a central role in digital transformation initiatives, yet its relatively limited influence compared with technological competence and management support is not sufficiently interpreted or explained.

18. Greater effort should be devoted to comparing the findings with prior BIM adoption studies.

19. Much of the literature review adopts a descriptive citation style where references are used to support definitions rather than build arguments, resulting in a theoretical foundation that feels more encyclopedic than analytical.

20. The proposed conceptual model does not consider potential mediating or moderating mechanisms, despite the likelihood that organizational and environmental factors influence outcomes through more complex pathways than those represented in the current framework.

21. Practical implications are presented at a very high level of abstraction, with recommendations directed simultaneously toward firms, policymakers, and urban planners despite the study only examining perceptions of construction professionals.

22. Numerous sections contain grammatical inconsistencies, awkward sentence construction, and tense shifts between future and past forms, suggesting that the manuscript would benefit substantially from professional language editing before publication.

23. Perhaps the most important concern is that the paper ultimately promises insight into BIM-enabled green building adoption and smart city transformation, yet the empirical evidence remains limited to self-reported perceptions collected through a single survey instrument, making several of the broader claims appear considerably stronger than the data can realistically support.

Reviewer #2: The authors are encouraged to carefully verify that all cited references accurately support the statements and claims made throughout the manuscript. At present, several problem statements and arguments appear to be supported by references that are not directly relevant to the issues being discussed. This weakens the justification for the study and raises concerns regarding the robustness of the literature foundation.

The authors should thoroughly review the cited literature and ensure that each reference is appropriately aligned with the corresponding claim. Strengthening the linkage between the problem statements and the supporting evidence is necessary to improve the credibility of the study. Until these issues are adequately addressed, the reliability of the findings and conclusions remains uncertain.

Reviewer #3: Reviewer report

PONE-D-26-24632

Title: BIM-Supported Green Building Adoption in the Construction Industry

The authors states that data will be available from the corresponding author after reasonable request. If full data availability is part of the requirement of the journal, the authors are advised to make the data available as part of the open access policy of most publishers.

The authors should briefly state how ethics were followed in this study.

It is not clear where the variables and construct were derived from. The questionnaire could show this and possibly a new Table in the literature review section showing the items, category or constructs, indicators or variables, literature sources, and other importance details. These items should be summarised in a Table and provided in the LR section or appendix. The Questionnaire is not sufficiently Developed. The manuscript states that each construct was measured with five items but does not adequately describe: Item sources, Scale adaptation procedures, Content validity process, Pilot testing, and Expert review. Please the authors should try and provide (1) Complete measurement items in an appendix, (2) Original literature sources for each construct, and (3) Evidence of pilot testing and content validation.

Since all the variables were collected at the same time from the same experts using the survey approach, there is the tendency to have common method bias (CMB). Which must be assessed. You could use collinearity VIF report from PLS-SEM or Harman’s single-factor test from EFA or Marker variable approach and discuss the results.

Interesting analysis them but the report on the structural model is Incomplete. The paper reports path coefficients and significance values but omits several critical PLS-SEM outputs. Please look out for this Missing information that should be included in the report (R² values, Adjusted R², Effect sizes (f²), Predictive relevance (Q²), Model fit indicators (SRMR), and Multicollinearity diagnostics (VIF)).

There is an observed Conceptual Model Inconsistency. Seven hypotheses were proposed, however is is not clear how (H7 states BIM adoption contributes to smart and sustainable city development) was met or tested. The results section primarily reports determinants of BIM adoption. Statistical evidence for H7 is not clearly presented. If possible, the authors should indicate clearly in the paper such results as (1) Path coefficient, (2) t-value, (3) p-value, (4) R² ; for the BIM → Smart City Development relationship (H7).

There is a Weak Theoretical Contribution that should be strengthened. The study largely confirms relationships already established in technology adoption literature. The manuscript claims theoretical contribution through TOE–DOI integration. However, it does not clearly explain (1) Why integration is necessary, (2) What new theoretical insight emerges, and (3)How findings extend existing BIM adoption literature.

Consider reviewing the citations/References, as several references appear unrelated to BIM, green buildings, or construction management. These citations appear disconnected from the study topic and could impact citation quality and literature grounding.

Please avoid or minimize repetition and redundancy. In the paper, there are several sections repeating nearly identical ideas. e.g., Technological competence is defined twice in highly similar wording. See also the discussions on external support and DOI contain repetitive explanations. The authors could consider condensing the literature review to remove duplicated text.

The use of purposive sampling is not a problem. Even though it is allowed in exploratory study, the authors should consider discussing the potential biases associated with its use and how the approach limits sample representativeness and non-response biases. The discussion should state how these issues were addressed or minimised.

The study is limited to Saudi Arabia, yet conclusions generalised the findings to the broader construction industry. Please, try and restrict claims to the Saudi Arabian context or justify broader applicability.

Tone down on the claim of causality. There is an overstatement of causality in this study that uses cross-sectional survey data. Words like "influences," "drives," and "contributes" convey causality. Authors should consider words like "is associated with," "predicts," "is positively related to," and others.

What are the managerial and practical implementation effects of your findings? While statistical significance is well demonstrated, the authors dis not discuss practical significance of key results

Address the numerous grammatical and syntax issues throughout the paper.

Consider engaging a professional English-language editor.

**********

what does this mean?). If published, this will include your full peer review and any attached files.

If you choose “no”, your identity will remain anonymous but your review may still be made public.

**Do you want your identity to be public for this peer review?**  For information about this choice, including consent withdrawal, please see our Privacy Policy

Reviewer #1: No

Reviewer #2: No

Reviewer #3: **Yes:**  Emmanuel Eze

**********

[NOTE: If reviewer comments were submitted as an attachment file, they will be attached to this email and accessible via the submission site. Please log into your account, locate the manuscript record, and check for the action link "View Attachments". If this link does not appear, there are no attachment files.]

To ensure your figures meet our technical requirements, please review our figure guidelines: https://journals.plos.org/plosone/s/figures

You may also use PLOS’s free figure tool, NAAS, to help you prepare publication quality figures: https://journals.plos.org/plosone/s/figures#loc-tools-for-figure-preparation.

NAAS will assess whether your figures meet our technical requirements by comparing each figure against our figure specifications.

# Author comment: Author response to Decision Letter 1

**DOI:** 10.1371/journal.pone.0356894.r002

26 Jul 2026

Dear Reviewers,

Thank you so much for your guidance! I would like to let you know that we have carefully considered all the comments, and we truly hope that our revisions will meet your expectations. At the same time, we remain open to making any further improvements you may request. Thank you again for helping us enhance the quality of this manuscript!

Please see our responses in the attached file.

Best regards,

Attachment Submitted filename: Response to Reviewers PLOS (1).docx

# Peer review: Decision Letter 1

**DOI:** 10.1371/journal.pone.0356894.r003

**Authors:** Peng Zhang

10 Aug 2026

BIM-Supported Green Building Adoption in the Construction Industry

PONE-D-26-24632R1

Dear Dr. Alawag,

We’re pleased to inform you that your manuscript has been judged scientifically suitable for publication and will be formally accepted for publication once it meets all outstanding technical requirements.

Within one week, you’ll receive an e-mail detailing the required amendments. When these have been addressed, you’ll receive a formal acceptance letter and your manuscript will be scheduled for publication.

An invoice will be generated when your article is formally accepted. Please note, if your institution has a publishing partnership with PLOS and your article meets the relevant criteria, all or part of your publication costs will be covered. Please make sure your user information is up-to-date by logging into Editorial Manager at Editorial Manager® and clicking the ‘Update My Information' link at the top of the page. For questions related to billing, please contact billing support.

If your institution or institutions have a press office, please notify them about your upcoming paper to help maximize its impact. If they’ll be preparing press materials, please inform our press team as soon as possible -- no later than 48 hours after receiving the formal acceptance. Your manuscript will remain under strict press embargo until 2 pm Eastern Time on the date of publication. For more information, please contact onepress@plos.org.

Kind regards,

Peng Zhang, Ph.D.

Academic Editor

PLOS One

Additional Editor Comments (optional):

Reviewers' comments:

Reviewer’s Responses to Questions

**Comments to the Author**

Reviewer #1: All comments have been addressed

Reviewer #3: All comments have been addressed

**********

2. Is the manuscript technically sound, and do the data support the conclusions?

Reviewer #1: Yes

Reviewer #3: Yes

**********

3. Has the statistical analysis been performed appropriately and rigorously?

Reviewer #1: Yes

Reviewer #3: Yes

**********

4. Have the authors made all data underlying the findings in their manuscript fully available?

The PLOS Data policy

Reviewer #1: Yes

Reviewer #3: Yes

**********

5. Is the manuscript presented in an intelligible fashion and written in standard English?

Reviewer #1: Yes

Reviewer #3: Yes

**********

Reviewer #1: Thanks my comments have been addressed. I look forward to reading the article in press. All the best

Reviewer #3: (No Response)

**********

what does this mean?). If published, this will include your full peer review and any attached files.

If you choose “no”, your identity will remain anonymous but your review may still be made public.

**Do you want your identity to be public for this peer review?**  For information about this choice, including consent withdrawal, please see our Privacy Policy

Reviewer #1: No

Reviewer #3: No

**********

# Editor report: Acceptance letter

**DOI:** 10.1371/journal.pone.0356894.r004

**Authors:** Peng Zhang

PONE-D-26-24632R1

PLOS One

Dear Dr. Alawag,

I'm pleased to inform you that your manuscript has been deemed suitable for publication in PLOS One. Congratulations! Your manuscript is now being handed over to our production team.

At this stage, our production department will prepare your paper for publication. This includes ensuring the following:

* All references, tables, and figures are properly cited

* All relevant supporting information is included in the manuscript submission,

* There are no issues that prevent the paper from being properly typeset

You will receive further instructions from the production team, including instructions on how to review your proof when it is ready. Please keep in mind that we are working through a large volume of accepted articles, so please give us a few days to review your paper and let you know the next and final steps.

Lastly, if your institution or institutions have a press office, please let them know about your upcoming paper now to help maximize its impact. If they'll be preparing press materials, please inform our press team within the next 48 hours. Your manuscript will remain under strict press embargo until 2 pm Eastern Time on the date of publication. For more information, please contact onepress@plos.org.

You will receive an invoice from PLOS for your publication fee after your manuscript has reached the completed accept phase. If you receive an email requesting payment before acceptance or for any other service, this may be a phishing scheme. Learn how to identify phishing emails and protect your accounts at https://explore.plos.org/phishing.

If we can help with anything else, please email us at customercare@plos.org.

Thank you for submitting your work to PLOS One and supporting open access.

Kind regards,

PLOS One Editorial Office Staff

on behalf of

Prof. Peng Zhang

Academic Editor

PLOS One
